# Evaluation of the Role of Functional Constraints on the Integrity of an Ultraconserved Region in the Genus *Drosophila*

**DOI:** 10.1371/journal.pgen.1002475

**Published:** 2012-02-02

**Authors:** Carlos Díaz-Castillo, Xiao-Qin Xia, José M. Ranz

**Affiliations:** 1 Department of Ecology and Evolutionary Biology, University of California Irvine, Irvine, California, United States of America; 2 Institute of Hydrobiology, Chinese Academy of Science, Wuhan, China; Duke University, United States of America

## Abstract

Why gene order is conserved over long evolutionary timespans remains elusive. A common interpretation is that gene order conservation might reflect the existence of functional constraints that are important for organismal performance. Alteration of the integrity of genomic regions, and therefore of those constraints, would result in detrimental effects. This notion seems especially plausible in those genomes that can easily accommodate gene reshuffling via chromosomal inversions since genomic regions free of constraints are likely to have been disrupted in one or more lineages. Nevertheless, no empirical test has been performed to this notion. Here, we disrupt one of the largest conserved genomic regions of the *Drosophila* genome by chromosome engineering and examine the phenotypic consequences derived from such disruption. The targeted region exhibits multiple patterns of functional enrichment suggestive of the presence of constraints. The carriers of the disrupted collinear block show no defects in their viability, fertility, and parameters of general homeostasis, although their odorant perception is altered. This change in odorant perception does not correlate with modifications of the level of expression and sex bias of the genes within the genomic region disrupted. Our results indicate that even in highly rearranged genomes, like those of Diptera, unusually high levels of gene order conservation cannot be systematically attributed to functional constraints, which raises the possibility that other mechanisms can be in place and therefore the underpinnings of the maintenance of gene organization might be more diverse than previously thought.

## Author Summary

Eukaryotic genomes have been reshaped by chromosomal rearrangements during evolution. However, the comparison of distantly related species has uncovered unusually large genomic regions with conserved gene organization. A widely accepted explanation is that, in those regions, there exist genes with joint and/or intricate regulation, which, if altered, might affect the performance of the carriers. We used a system that allowed us to precisely disrupt one of the largest genomic regions that has been conserved in the fruit flies since ∼63 million years ago and performed a variety of assays devised to detect differences in organismal performance and anomalous gene expression. Despite the overrepresentation of genes with expression profiles related to male fertility and detection of chemical stimuli in this genomic region, as well as the presence of genes expressed during development and putatively regulated by long distantly located sequences, we do not find evidence of diminished viability and fertility or of anomalous levels of expression. These results lead us to propose that regulatory constraints might not suffice to explain the maintenance of the integrity of some of the best candidate regions in one of the most dynamic eukaryotic genomes.

## Introduction

Collinearity conservation, *i.e.* conservation of local gene order, across distantly related phyla is often viewed as the result of functional constraints that prevent the occurrence of breaks of chromosomal rearrangements during evolution. The nature of these constraints is still poorly understood. They may merely reflect the presence of yet-to-be annotated protein and nonprotein-coding genes in intergenic regions [1]–[3]. A second type of constraints may be linked to the existence of regulatory domains, *i.e.* genomic regions associated with complex regulatory inputs. These regulatory domains can adopt at least two forms. One common form of complex regulatory inputs corresponds to genes that are coordinately expressed or repressed [4]–[7]. Alternatively, regulatory constraints can adopt the form of long-range regulatory interactions, which often involve the interdigitation of *cis*-regulatory sequences with genes that are not their targets [8]–[10]. These regulatory domains are enriched for noncoding DNA (highly conserved noncoding elements or HCNEs) with putative regulatory potential [11]–[16]. These HCNEs tend to be found in the vicinity of protein-coding genes that participate in key processes during development, such as regulation of gene expression and signal transduction [10], [12], [17]. Disruption of genomic regions under constraints can be accompanied by alteration of gene activity, as illustrated by chromosomal rearrangements that modify gene expression as a result of the separation of a gene from its regulatory sequences [18]–[20]. These alterations in gene activity may have a detrimental effect, which would lead to conservation of gene organization [19], [21].

Highly rearranged genomes, such as those of the Diptera, are especially suitable for analyzing the presence of regulatory-based constraints that preserve collinearity since regions free of them are likely to have been disrupted in one or more lineages. Gene order comparisons have helped to delineate collinear blocks [22] across nine *Drosophila* species that represent ∼380 million years (myr) of total divergence time [23], [24]. A minimum of ∼6,100 chromosomal breakpoints have been estimated to have occurred in 2,688 intergenic regions [22] scattered across the main chromosomal elements (the so-called Muller's elements A–E) that constitute the *Drosophila* genome [25], [26]. The analysis of the expected patterns of evolution of gene organization under different evolutionary scenarios indicate that fragile regions, *i.e.* those that accumulate most chromosomal breaks during evolution [27], are the main factor that explains the patterns of gene arrangement across *Drosophila* species [22]. Constraints nevertheless may be responsible for ∼15% of gene order conservation and their presence is positively correlated with the size of the collinear block [22]. The top 1% largest collinear blocks, or ultraconserved regions [22], are enriched for genes associated with particular expression profiles, but the functional signature most prominently found in ultraconserved regions are stretches of DNA with multiple HCNEs (14.5% of the 145 HCNE peaks mapped as compared to 6.7% expected).

To our knowledge, only two empirical tests for the presence of functional constraints have been performed in eukaryotic genomes [20], [28]. In both cases, engineered chromosomal inversions were induced to disrupt clusters of genes with shared expression attributes and, subsequently, the phenotypic consequences of such disruptions monitored. For example, the disruption of the mouse *Hoxd* cluster, which is conserved across vertebrates, helped determine the presence of two functional subdomains and two long-range enhancers on either side of the cluster. This functional organization of the *Hoxd* cluster was proposed as the underlying cause that kept the cluster intact during the evolution of vertebrate lineages. In Diptera, three gene neighborhoods expressed in testes and one gene neighborhood expressed during embryogenesis of *D. melanogaster* have been disrupted [28], but no modification of the expression of the genes included in the neighborhoods examined was detected. These neighborhoods are conserved within the *D. melanogaster* species subgroup but disrupted in some other *Drosophila* lineages (Table S1), which does not clarify the phylogenetic scope of the putative constraints tested. The phenotypic consequences of disrupting a collinear region conserved throughout the genus *Drosophila*, which is presumably maintained by constraints, are presently unknown.

**Figure 1:**
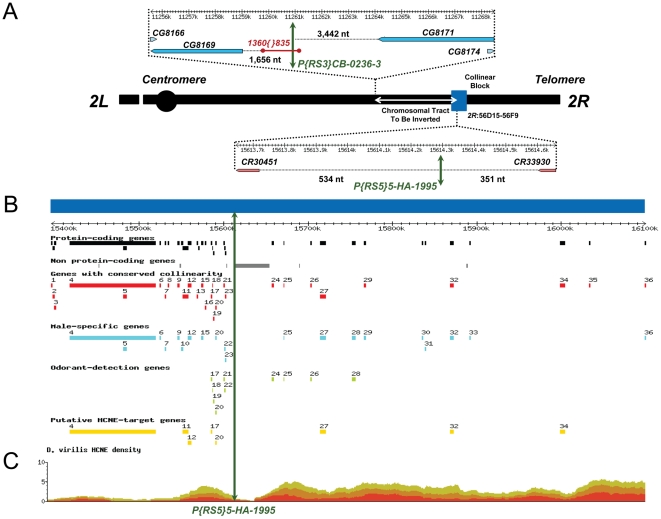
Schematic representation of the inversion engineered to disrupt the ultraconserved region *CG15121–CG16894* and its salient features. (A) Schematic representation showing the surrounding regions and location of the FRT-bearing TEs *P{RS5}5-HA-1995* and *P{RS3}CB-0236-3* (green double arrowhead lines) used to generate the inversion *In(2R)51F11-56E2* (white double arrowhead line), which disrupts the ultraconserved region *CG15121–CG16894* (blue box). The element *P{RS3}CB-0236-3* is inserted in a naturally occurring copy of the TE *1360{}835* [43]. Distances between the FRT-bearing TEs and the flanking genes are indicated in nucleotides. Notice that flanking transcription units at the immediate vicinity of the inner breakpoint are two non-protein-coding genes: *CR30451*, which codes for *tRNA:E4:56Fc*; and *CR33930*, which codes for *snoRNA:185*. (B) Annotation of the ultraconserved region *CG15121*–*CG16894* using *D. melanogaster* as a reference. From top to bottom: 36 protein-coding genes; 122 non-protein-coding genes (8 miRNAs, 13 tRNAs, 1 snoRNA, and 100 5S rRNAs); 29 protein-coding genes whose collinearity is maintained across nine species of the genus *Drosophila*; 20 protein-coding genes with male-biased gene expression using mRNA levels as a proxy; 10 protein-coding genes related to odor-guided behavior; and 8 putative targets of Highly Conserved Non-coding Elements (HCNEs) based on their expression profile, core promoter predictions, and mutant phenotypes. Details on the protein-coding genes in this region and their annotation are provided in Table S2. (C) Ancora [82] snapshot (http://ancora.genereg.net) of the distribution of HCNEs when genome sequences of *D. melanogaster* and *D. virilis* are compared. Green, orange, and red denote 96%, 98%, and 100% nucleotide identity, respectively.

Here, we used chromosome engineering to disrupt an ultraconserved region located on Muller's element C of the *Drosophila* genome (Figure 1A). This ultraconserved region is delimited by the genes *CG15121* and *CG16894* and is ∼701 kb long in *D. melanogaster* [22]. Importantly, this ultraconserved region is, based on current information, the one with the largest number of functional signatures, suggestive of the presence of regulatory-based constraints [22]. After disrupting the ultraconserved region *CG15121–CG16894*, we examined the resulting phenotypic consequences both by performing a variety of genetic and competition experiments that tested for differences in viability, fertility, and relevant parameters of general homeostasis and by characterizing levels of mRNA abundance in both sexes. Our results indicate that, contrary to the prevalent view, the extraordinary conservation of some of the largest collinear blocks in eukaryotic genomes might not necessarily derive only from functional constraints that result in severe detrimental effects.

## Results

### Functional and comparative organization of the ultraconserved region *CG15121–CG16894*

The ultraconserved region *CG15121–CG16894* ranked first in length and eleventh in the number of genes encompassed among 2,683 regions of conserved collinearity across nine *Drosophila* species [22]. Data from another comparative analysis on gene organization in the genus *Drosophila* [29] are consistent with the overall maintenance of the collinearity in this genomic region. In addition, this region shows statistically significant enrichment for genes encoding proteins involved in chemosensory perception and for genes preferentially expressed in males, many of them showing this same trend across multiple *Drosophila* species (Figure 1B and Table S2). This higher-than-expected local density of genes with coherent patterns of expression supports the presence of a male-biased gene expression neighborhood, which is intertwined with a smaller chemosensory perception gene neighborhood.

This ultraconserved region is spanned by four HCNE peaks [12] (Figure 1C), more than any other collinear block. Genes responsive to HCNEs have been postulated to be preferentially associated with a particular kind of core promoters. Specifically, using promoter predictions for 42% of the protein-coding genes of *D. melanogaster*, a significant overrepresentation of genes with some kind of Inr-motif (Inr only, Inr/DPE, or Inr/TATA) was found in HCNE-dense regions [12]. We screened 500 nt upstream of each protein-coding gene in the region under study using McPromoter and obtained reliable predictions for eight genes. Six of these genes were predicted to have a core promoter responsive to HCNEs. These genes are found scattered along the region (Figure 1B and Figure S1) and encode proteins involved in the regulation of gene expression, from mRNA binding proteins (*sm*), to signal transducers (*Toll-7, 18-w*), to ubiquitins (*Isopeptidase-T-3*), and also to others that we did not predict *a priori* (*Obp56a* and *CG8654*). Given the large number of genes for which a prediction was not provided by McPromoter, we checked for the presence of genes that are expressed during key developmental stages using the expression profile characterization generated by modENCODE [2]. Fifteen out of 36 protein-coding genes show a preferential pattern of expression during embryogenesis; among them, there is a cluster of eight genes mostly displaying high levels of expression during the first 16 h of development and moderate expression during the larva-pupa transition (Figure S1). Six of these eight genes are predicted to have core promoters of the Inr-motif type and four are associated with lethal phenotypes, the latter underscoring their functional relevance prior to imago emergence (Figure S1) [30], [31]. Together, these functional features associated with some of the genes in the region are suggestive of regulation by HCNEs.

Examination of the organization of the ultraconserved region *CG15121–CG16894* in *Anopheles gambiae* revealed the presence of orthologues in six different locations (Figure S2A and Table S3). This degree of dispersion is not surprising given the fast differentiation of the *Drosophila* and *Anopheles* genomes via the accumulation of chromosomal rearrangements [32]–[34] and the lack of common constraints reflected in the pronounced differences in development, morphology, and ecology of these two Diptera [33], [35], [36]. Nevertheless, we detected a conserved gene arrangement including the gene *Toll-7* and the *Obp* genes, an association that is also present in *A. aegypti* (Figure S2B). Phylogenetic analyses [37] unambiguously support the close phylogenetic relationship among several *Obp* genes in the ultraconserved region under study (*Obp*56a, *Obp56d*, and *Obp56e*) and some that are adjacent to *Toll-7* in *A. gambiae* (*OBP23*, *OBP25*, *OBP26*, *OBP28*), which in turn are closely related to those that cluster nearby *Toll-7* in *A. aegypti* ([38] and this work; Figure S2C). Interestingly, the genes *Obp*56a, *Obp56d* and *Toll-7* are found within the same expression cluster in *D. melanogaster* and two of them have core promoter types presumably responsive to HCNE-mediated regulation (Figure S1).

To sum up, the existence of two intertwined gene neighborhoods associated with very marked expression profiles, the enrichment for HCNE peaks and presence of their putative targets, and the detection of a region conserved across Diptera reinforces the possibility that one or more regulatory-based constraints might exist in the ultraconserved region *CG15121–CG16894*.

### Disruption of the ultraconserved region *CG15121–CG16894*

To assess the importance of the integrity of the ultraconserved region *CG15121–CG16894*, we aimed to disrupt it and characterize the resulting phenotypic effects. We examined the existence of stocks carrying isolated naturally occurring inversions disrupting the ultraconserved region and none was found. Thus, we generated a disruption of the ultraconserved region *CG15121–CG16894* by inducing the inversion *In(2R)51F11-56E2* in *D. melanogaster*, a species in which nonallelic homologous recombination (NAHR) events can be mediated between FLP recombination target-bearing transposable elements (FRT-bearing TEs hereafter) via activation of a heat-inducible flippase-recombinase [28], [39]–[42] (Figure S3, S4, S5). For that, we used two TEs bearing FRT sites in opposite orientation: *P{RS5}5-HA-1995*, which is inside the ultraconserved region *CG15121–CG16894*; and *P{RS3}CB-0236-3*, which is located 4.35 Mb upstream (Figure 1A). The outer element is virtually terminal within a collinear block of 15 genes and is inserted into a naturally occurring copy of the TE *1360{}835* [43].

**Figure 2:**
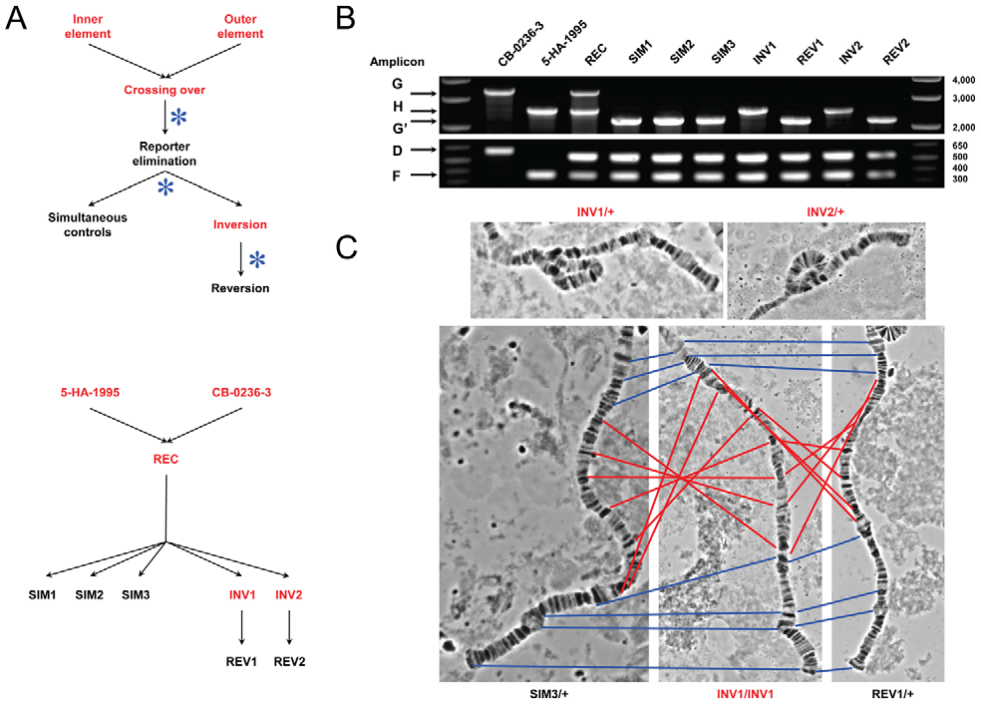
Disruption of the ultraconserved region *CG15121*–*CG16894*. (A) Outline of the main steps used to obtain the strains carrying the ultraconserved region *CG15121*–*CG16894* in its disrupted and intact form (Figures S3 and S4 for details). The strains 5-HA-1995 and CB-0236-3 carrying the FRT-bearing TEs [42] were used to induce the inversion *In(2R)51F11-56E2*, which disrupts the ultraconserved region (strains INV1 and INV2). Control strains carrying the ultraconserved region in its intact form are also shown (REC, SIM1-SIM3, REV1-REV2; Table S4). Black and red denote *w^−^* and *w^+^* phenotype, respectively; asterisk, heat-shock induced FLP expression. (B) Distinctive banding pattern for the PCR products of five different amplicons (D, F, G/G′, H) run simultaneously for each of the strains generated (Material and Methods, Figure S5, and Tables S5 and S6). Two µl from each individual PCR product were combined. Strains 5-HA-1995 and CB-0236-3 show the presence of only one of the FRT-bearing TEs in their genomes while the REC strain shows the presence of both. SIM and REV strains show the successful amplification of the amplicon G′, which denotes the presence of a derivative of *P{RS3}CB-0236-3* at the outer breakpoint, but not of the amplicon H, indicating that they carry the *2R* standard chromosomal arrangement. INV strains show the opposite pattern; the detection of amplicon H denotes the presence of the inverted chromosomal arrangement. (C) Representative polytene chromosome squashes. Top, an inversion loop is observed for the heterozygote progeny of INV1 and INV2 parentals when crossed with individuals from the strain *w^1118^* thus confirming the presence of the inverted arrangement. Bottom, no inversion loop is observed in the progeny of similar crosses involving SIM3 and REV1. Red and blue lines connect the same polytene band inside and outside of the inverted fragment, respectively, between carriers for the standard arrangement (SIM3 and REV1) and homozygotes for the induced inversion (INV1; center). Apart from the inversion *In(2R)51F11-56E2* in the strains INV1 and INV2, no other gross chromosome alteration was detected for the strains shown in (B).

We adopted several measures to avoid confounding effects that could overlay those of the intended disruption of the ultraconserved region *CG15121–CG16894*. First, TEs were selected to avoid disrupting any known regulatory sequences of flanking genes and those presumably embedded in HCNE peaks, thus preventing the generation of artifactual position effects. The comparison of the size and sex ratio of the progeny of flies homozygous for each of the TEs confirmed the absence of any obvious detrimental effect associated with particular TE insertions (Figure S6). Second, in addition to strains carrying the inversion *In(2R)51F11-56E2* (INV1 and INV2), we generated several control strains to account for further mutations that could have been incidentally generated by our approach [44]. Specifically, three kinds of control strains were constructed: strains carrying two FRT-bearing TEs in *cis* (REC), *i.e.* just before inducing the NAHR event that mediates the inversion; strains carrying the standard arrangement as a result of failed induced NAHR events but that were exposed to the same experimental conditions as the INV strains -SIMultaneous controls- (SIM1, SIM2, and SIM3); and strains in which the inverted segment is reverted back to its original orientation -REVertant controls- (REV1 and REV2) (Figure 2A, Figure S3 and S4). The main molecular changes at the inversion breakpoints of all relevant strains, plus those carrying the original FRT-bearing TEs, are depicted in Figure S5. In the absence of any secondary effect of our procedure, SIM, REV, and REC should perform likewise as measured by the size and sex ratio in the progeny of low-density crosses with homozygous flies (Figure S7). INV strains for which no SIM and/or REV control lines could be generated were discarded. For the remaining strains, their putative karyotype was verified at the cytological level and the expected molecular organization at their breakpoint regions confirmed by PCR and Sanger sequencing (Figure 2B–2C and 8). Further expression profiling confirmed the absence of local position effects at the inversion breakpoints (see below). Lastly, engineered chromosomes were maintained in homozygosis thus preventing the otherwise unavoidable accumulation of detrimental mutations if kept in heterozygosis over a balancer chromosome. Most of the strains that were not discarded were included in one or more downstream analyses.

### Phenotypic consequences of disrupting the ultraconserved region *CG15121–CG16894*

The disruption of the ultraconserved region *CG15121–CG16894* did not lead to viability impairment in the progeny of the carriers (INV1, INV2) as compared to that of non-carriers based on two proxies examined (Figure S7). Cursory examination of embryos, larva, and pupa did not detect any obvious morphological defect either. We then explored the possibility of a detrimental effect in heterozygous condition before reaching adult eclosion, either because of the disruption of the collinear block, meiotic distortion due to the presence of a chromosomal inverted rearrangement, or both. The comparison of different chromosome combinations revealed no departures from the expected Mendelian ratios (1∶2∶1) and absence of sex-specific effects (Figure S9). Further, we assessed differential viability during early stages (*i.e.* prior to imago emergence), when competition among individuals is specially intense [45], [46], and when most of the genes putatively targeted by HCNEs exhibit high levels of expression. Differences among carriers of different *2R* chromosomes were detected in frequency-dependent competition experiments involving different pairwise combinations of embryos, but those differences were not consistently shown by the carriers of the disrupted ultraconserved region (INV1, INV2) compared to those that carry it in its intact form (REC) across different genetic backgrounds (Figure S10). Together, these results do not point out to any obvious detrimental effect on viability, and therefore on stages of the life cycle that encompass key developmental transitions, as a result of disrupting the ultraconserved region *CG15121–CG16894*.

**Figure 3:**
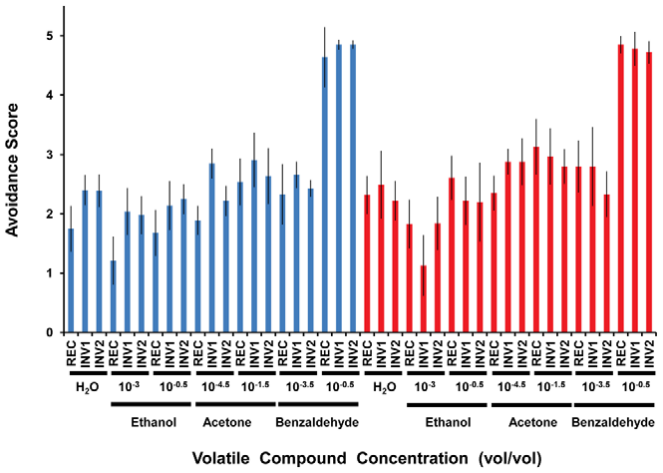
Olfactory response of strains with (INV1, INV2) and without (REC) the disrupted ultraconserved region *CG15121–CG16894* to three different volatile compounds. Blue, females; red, males. The “dipstick” method was used in all cases. An avoidance score equal to 2.5 indicates indifference while values <2.5 and >2.5 are interpreted as attractant and repellent responses, respectively. Ethanol, acetone, and benzaldehyde were assayed at two different concentrations (vol/vol) deferring by several orders of magnitude in order to test the response of the strains in very different conditions. Distilled water was used as a reference for the default response when no compound is added. For females, we found statistically significant differences (Kruskal-Wallis, d.f. = 2 in all cases) across strains in response to distilled water (*P*<0.0107), ethanol (concentration: 10^−3^, *P*<0.0056), ethanol (concentration: 10^−0.5^, *P*<0.0230), and acetone (concentration: 10^−4.5^, *P*<0.0002), while in males, the differences were confined to ethanol (concentration: 10^−3^, *P*<0.0183) and acetone (concentration: 10^−4.5^, *P*<0.0182). INV1 and INV2 showed significant differences in the same direction in relation to REC after accounting for multiple tests in the case of distilled water (females), ethanol (concentration: 10^−3^, females), and acetone (concentration: 10^−4.5^, males) (Table S14). Error bars indicate 95% CI.

Next, we examined the effect of disrupting the ultraconserved region *CG15121–CG16894* after imago emergence since many of the protein-coding genes included in the region under study are expressed during adulthood, often in a sex-dependent fashion (Figure S1). Specifically, the region under study is populated with male-biased genes in expression, 11 of them preferentially expressed in testes [22] and four, all of them *Obp* genes, present in the seminal fluid (Figure 1; Table S2). We tested for differences in several parameters of male fertility: progeny size, sperm performance, and mating ability. The comparison of males from the strains INV1, INV2, and REC, which are all red-eyed so that differences due to differential pigmentation can be factored out, revealed that although there are differences in progeny size (Figure S11A), strains INV1 and INV2 are more different from each other than either of them is to strain REC. Double-mating experiments did not reveal any substantial difference in sperm performance (Figure S11B) and mating ability (Figure S12). Further, given the presence of nine odorant-binding and one odorant-receptor protein-coding genes in the ultraconserved region *CG15121–CG16894*, we analyzed the odorant abilities of the different strains. We examined the response to three volatile compounds (ethanol, acetone, and benzaldehyde) and found statistically significant differences in five out of 12 sex-by-strain combinations. Importantly, for females exposed to ethanol at a concentration of 10^−3^ and males exposed to acetone at a concentration of 10^−4.5^, strains INV1 and INV2 exhibited a coherent pattern of differentiation from strain REC (Figure 3; Table S14), involving in all cases an attenuated attraction to the chemical in question. This attenuated response does not result from an overall impairment of the odorant abilities of the flies as shown by the response to the repellent compound benzaldehyde. Analyses of different proxies for the general adult homeostasis (negative gravitaxis, heat-shock resistance, desiccation resistance, and starvation resistance) did not uncover any other difference between the strains with and without the disrupted ultraconserved region *CG15121–CG16894* (Figure S13).

**Figure 4:**
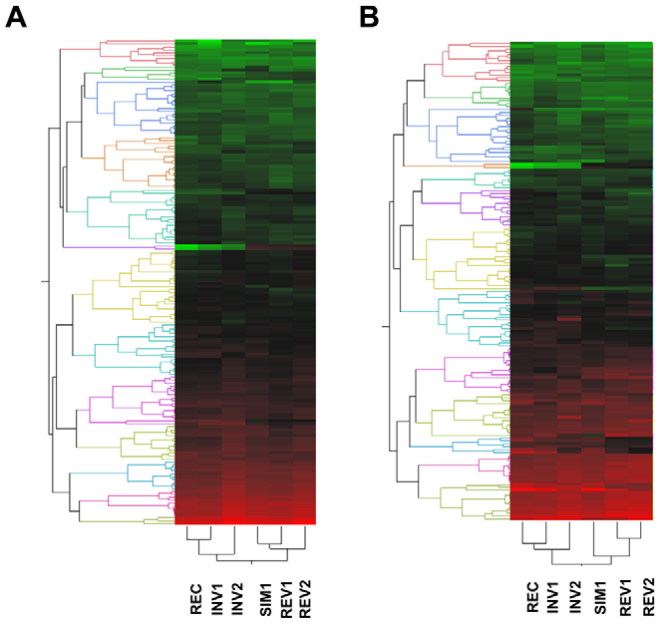
Two-way hierarchical clustering of the average levels of expression estimated for the 1% transcripts exhibiting the lowest *P* values across the six strains under study according to one-way ANOVA. (A) Males; (B) females. For both sexes, the patterns of expression among red-eyed strains, *i.e.* those with (INV1, INV2) or without (REC) the disrupted ultraconserved region *CG15121–CG16894*, are more similar to each other than either of them is to any of the white-eyed strains (SIM1, REV1, REV2), all of them carrying the ultraconserved region in intact condition. Thirty-six one-color hybridizations were performed involving three biological replicates for each sex-by-strain combination. Red, high expression; green, low expression.

Lastly, we tested whether the disruption of the ultraconserved region *CG15121–CG16894* resulted in a perturbation of gene expression. We performed a microarray-based characterization of the transcriptome of six lines (REC, INV1, INV2, SIM1, REV1, and REV2) during adulthood, the stage in which we found evidence of phenotypic differences associated with the disruption generated. At FDR 0.01, we found a very limited number of differentially expressed transcripts both in males and females (0.07% -11/16,637- and 6.2% -1,033/16,637-, respectively; Dataset S1 and Table S16). Further analyses confirmed the similarity of the expression profiles between equivalent strains generated by our procedure (first three planned contrasts in Table S17; Datasets S1 and S2). Likewise, these analyses indicated that the most statistically significant differences in mRNA abundance found are associated with differences in pigmentation (last three planned contrasts in Table S17; Dataset S2), in good agreement with the clustering of expression profiles among strains (Figure 4). The inspection of the chromosomal distribution of the differences in gene expression showed that a few of them were related to genes in the ultraconserved region. However, these alterations in mRNA levels are in fact the result of pigmentation differences, given that these alterations were found invariably between red-eyed and white-eyed strains, regardless of whether the former carry the disrupted (INV1 and INV2) or the intact form (REC) of the ultraconserved region (Figures S14 and S15). Searches for biologically coherent patterns among differentially expressed genes indicated that, for example for females, statistically significant enrichment was found for functional classes related to perception of visual stimuli (Table S18). Further, patterns of sex bias in gene expression were not affected either (Figure S16; Dataset S3). Hence, no discernible effect on the levels of mRNA of genes both inside and outside of the ultraconserved region *CG15121–CG16894* was detected as a result of its disruption.

## Discussion

The interplay among organization, function, and evolution of eukaryotic chromosomes is still poorly understood. Collinearity conservation is an important genomic feature that can conflate all three aspects, especially in genomes characterized by their ease in accommodating structural variation. Up to date, no empirical evaluation has been performed on the effects of disrupting the integrity of one of the largest genomic regions whose overall gene organization has been preserved over a large time scale. Importantly, this conservation entails species with very different behaviors and ecologies and therefore subject to very different selective pressures [46], [47]. Our results show an absence of detrimental effects on the carriers of the disrupted ultraconserved region for a variety of traits associated with viability and fertility. Although the absence of detrimental phenotypic effects could result from a limited ability to detect differences as statistically significant, the comparison of actual and estimated ideal samples sizes shows that this potential limitation could be the explanation in only a few cases (Table S19). Therefore, at least in Diptera genomes, our results show that an unusually high degree of collinearity conservation coupled with enrichment for functional coherent patterns is not necessarily associated with severe detrimental effects upon perturbation [20], [48].

The ultraconserved region *CG15121–CG16894* harbors at least three sets of protein-coding genes that may be associated with regulatory-based constraints. The genes fall into the following broad categories: detection of chemical stimuli; sperm manufacturing and performance; and developmental processes. The only phenotype detected in association with our disruption was the more attenuated odorant response to attractant volatile compounds. Although variation in olfactory response occurs both within and between species [49], [50], it has been shown that the altered function of *Obp* genes, including some in the region studied, can affect fitness components in *D. melanogaster* [51]. Transcriptome characterization of adult flies did not uncover any obvious consequences in the activity of the *Obp* genes due to our disruption as measured by mRNA abundance, and magnitude and direction of sex bias. This lack of evidence for misregulation cannot rule out though that some other expression attributes, such as the spatial distribution of transcripts, had not been altered since they would have gone unnoticed by our approach. The evolutionary relevance of the maintenance of the clustering of *Obp* genes throughout the genus *Drosophila* is reinforced by the presence of an orthologous arrangement in different mosquito species to an extent not explained by chance [37]. In fact, the maintenance of the arrangement that includes a core cluster of *Obp* genes and the gene *Toll-7* for ∼970 myr was unexpected due to the extent of rearrangement undergone by the genomes of the Diptera involved [22], [33], [34] (Figure S2B). Regardless, the conservation of the cluster of *Obp* genes and *Toll-7* would not explain the collinearity conservation observed elsewhere in the ultraconserved region under study.

In relation to the two other biological signatures suggestive of constraints, we detected no evidence that our perturbation resulted in a detectable phenotype. The genes included in the two intertwined gene expression neighborhoods of the ultraconserved region *CG15121–CG16894* do not show evidence of altered mRNA levels or malfunction that could result in impaired male fertility. Whether the constituent genes are under a tight coordinated regulation [52] that could affect the long-term stability of the region does not seem to be upheld by our results. This conclusion would be reinforced by the genus-wide lability shown by clusters of male-biased genes in expression [22]. The absence of misregulation, as measured by mRNA abundance, is consistent with the results obtained when *D. melanogaster* male-specific gene neighborhoods spanning over hundred of kb were disrupted [28].

Further, protein-coding genes presumably responsive to long-range regulation mediated by HCNEs during development are found scattered across the ultraconserved region *CG15121–CG16894* at both sides of the disruption. The lack of a detrimental phenotype could reflect that the region is a composite of several autonomous genomic regulatory domains, each of them under the control of a particular HCNE peak. Under this scenario, our disruption would have separated different genomic regulatory domains without affecting any long-range interaction between HCNEs and their targets. Nevertheless, marked autonomy among HCNE peaks should result in certain regionalization of the expression profiles, *i.e.* physically closer HCNE targets should exhibit more similar expression profiles. In fact, we find putative targets of HCNEs located at both sides of the disruption, such as *CG9854* and *CG8896*, showing very similar expression profiles (Figure 1 and Figure S1). If this similarity denotes the existence of long-range interactions, the latter are not associated with detrimental effects that explain the collinearity conservation. Alternatively, some degree of regulatory redundancy could exist at both sides of the disruption, which would explain the lack of phenotypic effect but not the collinearity conservation.

A different view of collinearity conservation entails factors other than functional constraints. DNA stretches with sufficient sequence identity can mediate NAHR events that give rise to chromosomal rearrangements [53]. Large collinear regions could be depleted of this type of sequences thus explaining why this ultraconserved region has maintained its integrity. A search for well-annotated sequences with the potential to mediate NAHR events [54] in the studied region across *Drosophila* species revealed that these sequences are present (Table S20). These sequences include ncRNA genes such as tRNAs [55], [56], rRNAs genes [57], [58], and snoRNAs [59]. Equivalent comparative analysis focusing on TEs, once properly annotated, will enable to test whether the region under study is particularly depleted for these sequences, which would decrease its propensity of being rearranged. Further, recent findings indicate that orthologous landmarks harboring genes that bind to the nuclear periphery are significantly larger than those that do not harbor any suggesting that particular intranuclear localizations might provide molecular environments associated with higher levels of genome stability [60]. The ultraconserved region *CG15121–CG16894* is known to establish some contact with the nuclear periphery [61]. Specifically, it contains at least five protein-coding genes (*CG16716*, *CG13872*, *CG10822*, *CG8654*, *CG16898*) that exhibit statistically significant association with the B-type Lamin protein, a key component of the inner nuclear membrane [60], [62]. This pattern leaves open the possibility that some regulatory-based constraints had evolved under the enhanced evolutionary stability enjoyed by genomic regions associated with the nuclear periphery, and therefore functional constraints would not be the only mechanism contributing to their collinearity conservation.

Regardless how extensive the characterization of individuals carrying engineered genomic regions could be, our ability to detect phenotypic effects will always be contingent to the experimental setting used and the timescale in which the assays are performed. Nevertheless, our results raise the possibility that, at least in Diptera genomes, the mechanistic basis of collinearity conservation might be much more subtle and diverse than previously thought and that regulatory-based interactions might not suffice to account for the patterns of extensive conservation seen in some genomic regions [22]. Only further empirical tests for this and other ultraconserved regions can shed light on the scope of our observations.

## Materials and Methods

### Fly husbandry

Table S4 describes the strains used. Fly cultures were grown and maintained on dextrose-cornmeal-yeast medium at room temperature. Unless otherwise stated, all phenotypic assays were performed at 25°C in a temperature-controlled chamber and fly manipulation, sorting, and scoring were carried out under CO_2_ anesthesia. Strains generated are available upon request.

### Inversion generation

Strains carrying the original FRT-bearing TE insertions (5-HA-1996 and CB-0236-3) were selected from the DrosDel collection and examined at different levels prior to their use in the generation of the chromosomal inversion *In(2R)51F11-56E2*. Sanger sequencing confirmed the insertion point of the FRT-bearing TEs used. Low-density crosses using homozygous flies were also performed to test for potential detrimental effects associated with the TE insertions. Briefly, six sexually mature individuals (three four-day-old females and three two-day-old males) were used per cross and strain; each cross was replicated five times. Males were discarded after 24 hours while females were transferred twice every three days until discarded on the ninth day.

To generate the inversion *In(2R)51F11-56E2*, we followed essentially [42] with slight modifications (John Roote, pers. comm.; Figure S3A–S3B). Three types of strains are generated under this procedure (REC, INV, and SIM); a fourth type of strain (REV; Figure S3C) was generated by reverting the inversion *In(2R)51F11-56E2* [40]. The strains generated were scrutinized for evidence of side effect associated with our procedure (see below). Individuals from strains deemed as valid were crossed with those from the strain *w^1118^*, which possesses the standard arrangement for all chromosomes, and the third-instar salivary gland polytene chromosomes of the progeny examined. Chromosome squashes were stained with orcein and inspected with a Zeiss AX10 Imager M1 microscope. Cytological analysis was performed using the photographic polytene maps of *D. melanogaster* as a reference [63]. Diagnostic DNA stretches at the breakpoint regions were PCR amplified and their identity verified by Sanger sequencing; sequences were deposited at GenBank (http://www.ncbi.nlm.nih.gov/genbank/; JN805541–JN805602). Amplicons C, D, E, and F confirmed the presence of FRT-bearing TEs and their derivatives at the breakpoint regions. Amplicon G, or G′ after excision of one of the exons of the reporter gene, and H correlate with the absence and presence of the inversion, respectively (Tables S5 and S6 for further details on primers used and amplicons). Genomic DNA used in PCR genotyping was extracted from 50 individuals of each strain as described [39] and quantified using a NanoDrop 8000 Spectrophotometer. Takara Taq and Takara Ex Taq, depending on the size of the DNA fragment to be amplified, were used according to manufacturer conditions. PCR products were resolved on 1% agarose gels and visualized in an AlphaImager HP system. Amplicon sequences were examined for point mutations and indels that could have been generated incidentally during the course of our procedure.

### Viability tests

Low-density crosses using homozygous flies were set up for each strain, as described above for the evaluation of the TE insertions, to confirm absence of differences among control strains and to test for differences among the latter and the strains carrying the disrupted ultraconserved region. Additional crosses, five per strain, evaluated the effects of the disruption in heterozygous condition with different chromosomes carrying the standard arrangement (SIM1, REV1, REV2); 10 sexually mature individuals of each sex were used per cross. Further, adapted frequency-dependent experiments [64] evaluated differences in viability of mixtures of embryos for two different genotypes. Two types of mixtures were prepared. In both, *w^+^* individuals (INV1, INV2, or REC; the tested chromosome) compete with *w^−^* individuals carrying the ultraconserved region in its intact form. The only difference between the mixtures was the condition of the tested chromosome (heterozygosis, *e.g.* INV1/SIM1 versus SIM1/SIM1; homozygosis, *e.g.* INV1/INV1 versus SIM1/SIM1). For both types of mixtures, three sets of experiments were done varying the standard arrangement of the competing embryo (SIM1, REV1, REV2; the tester embryo), which was always in homozygosis. Embryos were collected from grape juice-sucrose-agar plates supplemented with yeast paste using FlyStuff Small Embryo Collection Cages. Using dissecting needles, 100 embryos were deposited on small grape juice-sucrose-agar cube according to one of three starting proportions for the two competing embryos (1∶3, 1∶1, 3∶1). The cube was subsequently introduced into a vial of fresh medium; each assay was replicated 10 times. Separate previous pilot experiments for the strains REC, SIM1, and INV1, using 100 embryos, indicated that the rate of survival was approximately 40% and therefore enough to detect differences between the strains to be compared; each assay was replicated 10 times. In total, 42 different competition settings were set up (7 tester by tested combinations×2 genotype conditions for the tested chromosome×3 starting proportions). Given the number of embryos used across competition experiments (42,000), only those involving the same tester *w^−^* embryo (*e.g.* SIM1/SIM1) were performed simultaneously, which determined how the contrasts were done (three for each genotype condition of the tested chromosome and particular starting proportion, *i.e.* 18 in total). No bias in sex ratio was assumed in all cases. Progenies from each cross were scored after 15 days to ensure the emergence of all surviving imagoes and the relative viability between the two competing genotypes estimated as (n_1_^′^×n_2_)/(n_1_×n_2_^′^), where n_1_ and n_2_, and n_1_^′^ and n_2_^′^, are the number of embryos and imagoes, respectively, of the two competing genotypes. Values of relative viability were log2-transformed.

### Male fertility tests

The effects of the induced disruption on male fertility were assayed by examining progeny size, sperm performance, and mating ability. For the first test, we exposed single four-day-old virgin females carrying the ultraconserved region *CG15121–CG16894* in its intact form (SIM1, REV1, REV2) to single two-day-old males with (INV1, INV2) or without (REC) the disrupted ultraconserved region. Males were discarded after 24 hours while females were transferred daily to vials with fresh food over a 10-day period. The number of replicates ranged from seven to nine. To evaluate sperm performance in the carriers of the disrupted ultraconserved region, we followed a similar experimental design to that for monitoring progeny size with the exception that the females mated in the first day were exposed to single males of their own strain in subsequent days. The progeny sired by the first and second males was scored based on eye color. For those days during which progeny from both parents were detected, *i.e.* those oviposited roughly the same day, we estimated the fraction of red-eyed individuals (necessarily sired by INV1, INV2, or REC males) in relation to the total. To account for the effect of the order of the males used, we performed identical experiments but this time the first male possessed the same genotype as the female while the second male was from the strains INV1, INV2, or REC. The number of replicates for each combination of genotypes ranged from four to nine. As for the comparison of mating abilities, we exposed single two-day-old males to 10 four-day-old virgin females for different time periods (1 hr, 3 hr, 6 hr). Afterwards, the females were transferred individually into vials with fresh food. After 15 days, 10 vials were examined for the presence of progeny, which indicates that at least one successful fertilization event occurred, and the number of females successfully fertilized recorded. Ten replicates were done per strain and time period combination.

### Response to volatile compounds

The “dipstick” method was used [65]. Briefly, virgin individuals from INV and REC strains were separated by sex and transferred by aspiration in groups of five to marked empty plastic vials (O.D.×H: 25×95 mm) 24 hr after emergence. The vials were marked at 3 and 6 cm from the bottom. Fisherbrand Q-tips dipped into the odorant dilutions to be tested were introduced into the vials up to the 6 cm mark, and secured with a cotton plug to avoid contact with the walls of the vial. After a 15-second recovery period, the number of flies in the bottom compartment was recorded 10 times every five seconds and the avoidance scored estimated as the average over those 10 measurements. A score of 2.5 indicates indifference to the odorant tested while values >2.5 and <2.5 indicate repulsion and attraction, respectively. All tests were performed between 2 and 6 pm after starving the flies for no less than four hours, and the vials were always placed sideways to prevent interfering with the geotactic response. For each strain, 10 groups of five individuals for each of the sexes were analyzed. We tested three odorants, two of them usually considered to elicit an attracting response (ethanol, Gold Shield; acetone, Fisher Chemical) while the other is considered to be a repellant (benzaldehyde; Sigma Aldrich B1334 Benzaldehyde-ReagentPlus). Since odorant response can be concentration dependent, we tested two concentrations deferring by several orders of magnitude. These concentrations (vol/vol) were: ethanol, 10^−3^ and 10^−0.5^; acetone, 10^−4.5^ and 10^−1.5^; and benzaldehyde, 10^−3^ and 10^−0.5^. Only fresh and thoroughly mixed dilutions were used to prevent oxidation, which is particularly relevant in the case of benzaldehyde. As a control, we used distilled water, which is known to attract starved flies [65].

### General homeostasis tests

Four proxies, including gravity response and survival to three stressors (heat-shock, desiccation, and starvation), were assayed. In all cases, males and females were separated after emergence and left for 24 hours in vials with fresh food. Subsequently, all individuals were transferred to new vials by aspiration for performing the pertinent tests. Negative gravitaxis was measured essentially as reported [66]. Twenty flies per sex and strain were transferred individually into a 250 ml glass volumetric cylinder. Flies were knocked down by tapping the cylinder ten times on a pad, and the height reached by each fly in 20 s recorded using the volumetric scale, which was divided by the maximum height. In our experience, flies performed more consistently after some training, reason why only measurements from a third trial were recorded. For the heat-shock resistance test [67], five groups of 20 flies per sex and strain were transferred in pairs into 3 ml Pyrex vials and then incubated in water baths at 35°C for 30 m (as a pre-conditioning step) and then at 39°C for another 30 m. The mobility of the flies was restricted to the submerged portion of the vial with cotton plugs. After the heat-shock treatment, flies were collected in vials with food that had been incubated at 25°C overnight. Next morning, the fraction of flies alive within each group was recorded. For the desiccation resistance test [68], groups of five flies, from a total of 20 per sex and strain, were transferred into empty vials. The mobility of the flies was restricted to the lower third of the vial using foam plugs, over which 3 g of Drierite desiccant were added. Next, the vials were sealed with Parafilm to maintain low humidity. Flies were checked every hour and the elapsed time-to-death recorded. As for the starvation resistance test, 20 flies per sex and strain were transferred into glass vials containing 2 ml of 1% agar dissolved in water to ensure normal humidity conditions. Flies were examined every 12 hr and the elapsed time-to death recorded.

### Statistical analysis of non-molecular phenotypes

For each phenotype, normality of the data was visually inspected using normal quantile plots and precisely determined with the Shapiro-Wilk test. Homogeneity of variances was estimated using the Levene's test. Parametric tests were used if the departure from the assumptions of normality and homoscedasticity was absent or negligible. The Welch ANOVA was used if only heterodasticity was detected. Different transformations (log2 or arcsine square root, depending on the test) of the measurements were calculated for some phenotypes to improve fit to normality although this had a very little effect. Alternatively, non-parametric tests (*e.g.* Kruskal-Wallis) were used. When multiple post-hoc contrasts were necessary, appropriate tests that account for multiple comparisons were used (*e.g.* Steel-Dwass). In the case of the departure from expected Mendelian ratios in frequency-dependent competition experiments, the *G*-test for goodness of fit, upon applying the William's correction, was used. Statistical contrasts were performed with JMP 4.1 (SAS Institute); the evaluation of the sensitivity to detect statistically significant differences was done with GPower 3.1.3 when needed [69].

### Expression data and analysis

Three low-density crosses were set up for the strains REC, INV1, INV2, SIM1, REV1, and REV2. The resulting virgin progeny was collected, separated by sex, and allowed to age for 5–7 days. Fifty individuals for each sex were snap frozen in liquid nitrogen at the same time of the day within a 2 hours window and subsequently stored at −80°C. Total RNA from biological samples was extracted using TRIzol Reagent (Invitrogen) and purified using the RNeasy Mini Kit (Qiagen). Concentration, quality and integrity of the RNA samples were estimated using a NanoDrop 8000 Spectrophotometer and the RNA 6000 Nano Chip Kit (Agilent Technologies) with an Agilent 2100 Bioanalyzer. Ten µg of total RNA were reverse transcribed into cDNA using the SuperScript Double-Stranded cDNA Labeling Kit (Invitrogen). Probe labeling, hybridization, array scanning, and data extraction were performed by Roche NimbleGen Service Group in Iceland. We used the oligonucleotide NimbleGen 12×135k *D. melanogaster* arrays, which contain 135,000 probes including different types of controls and 16,637 transcripts of protein-coding genes as annotated in release 5.7. The experiment consisted of 36 one-color hybridizations (6 samples×2 sexes×3 biological replicates). Raw fluorescence intensity values of probe pairs were summarized for each transcript using the median value after log transformation. Subsequent data analysis was performed using the tools implemented in the online pipeline WebArrayDB [70]. Data were normalized between arrays using the scale method [71] implemented in the LIMMA package [72]. Data for males and females were analyzed separately. Statistically significant differences across strains were assessed using a fixed-effect model ANOVA and multiple testing was performed with the Benjamini-Hochberg correction [73]. Similarity in expression profiles across genes and strains was assessed by hierarchical clustering using Ward's minimum variances as a distance metric. The first principal component was used to assist in the sorting. Six biologically meaningful planned contrasts defined a priori, all of them orthogonal, were done likewise by pooling the appropriately expression data of different strains as necessary (Table S17). Scrutiny of the differences in post-hoc comparisons among strains was done using the “multcomp” R package [74]. Functional information for relevant genes was obtained from FlyBase [30] and enrichment for particular Gene Ontology term categories (biological processes, molecular functions, and cellular components), KEGG pathways, and InterPro protein domains was evaluated using DAVID [75]. Benjamini-Hochberg correction [73] was applied to account for multiple tests. We proceeded likewise, but for each strain separately, to evaluate statistically significant differences between the sexes. Raw microarray data were deposited at the Gene Expression Omnibus database (GSE31120). In relation to the characterization of the expression profiles of the genes in the region under study during the life cycle of *D. melanogaster*, RPKM expression values across 30 timepoints and conditions [2] were extracted from FlyBase [30], log transformed, and compared using hierarchical clustering as above.

### Promoter analysis

Core promoter predictions for all the transcripts of protein-coding genes examined were done with McPromoter using the most stringent parameter values [76]. We inspected 500 nt upstream of the 5′ UTR start of each transcripts as annotated in FlyBase [30]. In the absence of an annotated 5′UTR, a stretch of DNA of equal length upstream of the first nucleotide annotated was examined. Categorization of genes as responsive to HCNEs was based on the prediction of having an Inr core promoter type.

### Comparative organization of the ultraconserved region *CG15121–CG16894*

We retrieved mapping coordinates of protein-coding genes included in the ultraconserved region under study in *A. gambiae* (AgamP3 assembly) using Biomart [77]. Any orthologous mapping information that did not conform to a one-to-one relationship between species was discarded. The global gene organization nearby the ortholog of *Toll-7* in *A. gambiae* and *A. aegypti* was examined through VectorBase [78]. Phylogenetic relationships among *Obp*-related amino acid sequences encoded by genes in the same region that harbors the gene *Toll-7* from the two mosquito species were conducted in MEGA 5.0 [79]. Amino acid sequences were downloaded from VectorBase [78] and aligned with CLUSTALW [80]. The best evolutionary model of amino acid evolution was found to be WAG and the consensus tree was built using the Maximum Likelihood method [81]. A discrete Gamma distribution was used to model evolutionary rate differences among sites (+G; 4 categories). Bootstrapping was performed to determine the confidence of the branches (1,000 replicates).

## Supporting Information

Dataset S1 Estimates of the average level of expression for all protein-coding genes in the *D. melanogaster* genome across six strains plus the statistical significance of differences in expression.
(7Z) Click here for additional data file.

Dataset S2 Statistical significance of differences in gene expression in relevant planned contrasts.
(7Z) Click here for additional data file.

Dataset S3 Statistical significance of differences in gene expression between males and females for six strains.
(7Z) Click here for additional data file.

Figure S1 Hierarchical clustering of the expression levels of the protein-coding genes included in the ultraconserved region *CG15121–CG16894* during the life cycle of *D. melanogaster*. Differences in expression levels are color coded (high expression, red ; low expression, green). Asterisks denote genes for which lethal phenotypes have been reported [30], [31]. Predictions for the type of core promoter were obtained using McPromoter [76]. The relative order of the protein-coding genes within the region under study is indicated as in Figure 1 from centromere to telomere. Common names for some genes are indicated in parentheses.
(TIF)

Figure S2 Comparative organization of the ultraconserved region *CG15121–CG16894* in Diptera. (A) Chromosomal location (red arrowhead) of six protein-coding genes with reliable one-to-one orthologous relationships between *D. melanogaster* and *A. gambiae* [77]. The same numerical code as in Figure 1 is used to indicate the identity of the gene. (B) Conserved collinearity of the gene *Toll-7* (blue) and *Obp* genes (red) across Diptera. Other intervening protein-coding genes are indicated in grey. *Drosophila* and *Anopheles* diverged ∼250 mya [83], *Anopheles* and *Aedes* diverged ∼150 mya [84], and the divergence time accumulated by the nine *Drosophila* species previously analyzed was ∼381 my [22]–[24], so that the total divergence time between the *Drosophila* and mosquito species considered is ∼970 my. Genes *Obp56f* and *Obp56i* are not indicated since they are not present in all *Drosophila* species examined [22], [85]. Doted lines indicate orthologous relationships ([38] and this work). Gene sizes and distances are not to scale in *D. melanogaster*. (C) Phylogenetic relationships of the OBP protein sequences encoded by genes nearby *Toll-7* between *A. gambiae* and *A. aegypti*. The percentage of replicate trees in which the associated taxa clustered together in the bootstrap test (1,000 replicates) is shown next to the branches when higher than the cut-off value of 0.5.
(TIF)

Figure S3 Crossing scheme followed to generate the inversion *In(2R)51F11-56E2*. (A) Generation of individuals carrying two FRT-bearing elements in *cis* (REC) upon recombination in the F2. (B) Generation of strains with (INV) or without (SIM for SIMultaneous control) the inversion *In(2R)51F11-56E2* using a heat-inducible flippase-recombinase. Notice that both strains derive from progeny generated from the same vial and therefore they have been exposed to the same experimental conditions. Grey box, mosaic flies for the inversion *In(2R)51F11-56E2*, both in the soma and the germ line. Carriers of the inversion are red-eyed (*w^+^*) and therefore readily identifiable. (C) Restoration of standard gene order via a heat-shock inducible reversion (REV for REVertant control). Only relevant chromosomes are indicated for the genotypes. Dotted box, genotype of flies subsequently made homozygous to construct the stocks to be used in further experiments. For clarity, the terminology used here in relation to FRT-bearing TEs and their derivatives in Table S4 is replaced by explicit indications on the number of FRTs and the state of the reporter gene. *2FRT*, original TEs (Figures S4 and S5 for further details).
(PDF)

Figure S4 Overview of the chromosomal changes occurred during the generation of the inversion *In(2R)51F11-56E2* and the corresponding eye phenotypes. Upon a crossing over event, two starting FRT-bearing TEs are placed on the same homolog. In the presence of a FLP recombinase source, recombination events are heat-shock induced between FRT sequences. The first FLP-mediated recombination event occurs between the two FRT sequences located within each TE leading to two recombined FRT sequences, one at each breakpoint, in opposite orientations. The second recombination event is mediated between these two resulting FRT sequences leading to the generation of the inversion. Phenotypic changes in the eye pigmentation of *Drosophila* adults are caused by alterations in the reporter gene mini-*white* carried by the TEs. These alterations occur as a result of the FLP-mediated FRT recombination events. Strains that carry a particular chromosome configuration are indicated. Details on the precise structure of the TEs in each strain are provided in Figure S5. Sizes and distances are not to scale.
(TIF)

Figure S5 Schematic representation of the molecular configuration of the FRT-bearing TEs at the breakpoint regions of different strains obtained in the course of the generation of the inversion *In(2R)51F11-56E2*. Two TEs bearing FRT sites in opposite orientation, *P{RS3}CB-0236-3* and *P{RS5}5-HA-1995*, were selected to generate a Type 1 Inversion according to the nomenclature in [42]. Notice that the two FRTs are flanking one of the exons of the modified reporter gene mini-*white* [86]. While the TEs are intact in the REC strain before the first heat-shock pulse, they undergo different kinds of molecular rearrangements during the rest of the protocol. Specifically, the recombination between the internal FRTs (yellow arrowhead) of each TE leads to the deletion of one of the exons of the reporter gene mini-*white* (orange and red boxes), which impairs its activity. If the second heat-shock pulse fails to induce a successful NAHR event between the single FRT present in each of the TEs, no rearrangement is generated and no reconstitution of the reporter gene occurs (SIM), which is associated with the *w^−^* phenotype. On the contrary, if ectopic recombination occurs, the newly generated inverted arrangement (INV) will be characterized by the presence of a reconstituted reporter gene at one breakpoint (and therefore by the *w^+^* phenotype) and one FRT at the other breakpoint [42]. A subsequent heat-shock pulse can lead to another successful ectopic recombination event restoring the original gene order (REV) and molecular organization at the breakpoint regions as before the inversion. The terminology used in for the FRT-bearing TEs and their derivatives follows that of Figure S4. Amplicons (A–H) used to confirm the molecular configuration of the breakpoint regions in all relevant strains are shown (Tables S5 and S6). Sizes and distances are not to scale.
(TIF)

Figure S6 Validation of strains carrying the FRT-bearing TEs used to generate the inversion *In(2R)51F11-56E2*. (A) Progeny size and (B) sex ratio (female to male) from low-density crosses of homozygous flies for each of the elements alone (5-HA-1995 and CB-0236-3), for both elements in *cis* (REC), and for flies with the same genetic background but carrying no transposable elements (*w^1118^*). No statistically significant difference was found among the strains (Kruskal-Wallis, d.f. = 3; progeny size, *P* = 0.0730; sex ratio, *P* = 0.8688; *n* = 5). Error bars indicate 95% CI.
(TIF)

Figure S7 Performance of the strains generated in low-density crosses with homozygous flies. (A) Progeny size and (B) sex ratio (female to male). Kruskal-Wallis test indicated that there are no statistically significant differences among strains both in progeny size (d.f. = 7; *P* = 0.2308) and in sex ratio (d.f. = 7; *P* = 0.0863). Pairwise contrasts confirmed that strains carrying the disrupted ultraconserved region *CG15121–CG16894* did not show significantly lower values than strains with the ultraconserved region in its intact form (Tables S7 and S8). Error bars indicate 95% CI (*n* = 5).
(TIF)

Figure S8 Sequence alignment of the region surrounding the FRT-bearing TEs *P{RS5}5-HA-1995* and *P{RS3}CB-0236*, and their derivatives across strains. (A) Outer (*2R*:11,260,347‥11,261,062) and (B) inner breakpoint (*2R*:15,613,890‥15,614,461), respectively, of the inversion *In(2R)51F11-56E2*. No major mutation was incidentally generated during the course of our experiments relative to the strain *w^1118^*, which was used by others to generate the strains of the DrosDel collection [41], [42]. The identity of the amplicons sequenced appears in parentheses (Table S6). The direct target sites duplications of the FRT-bearing TEs are easily identified in the region in which the two subsets of sequences overlap within each alignment. Sequence corresponding to TEs is not shown.
(PDF)

Figure S9 Average progeny size from seven heterozygotes that carry the ultraconserved region *CG15121–CG16894* in its disrupted (INV1, INV2) or intact (REC) form. Data for females and males are shown separately. The INV1, INV2, and REC chromosomes were tested in different combination with *2R* standard chromosomes associated with the *w^−^* phenotype (SIM1, REV1, REV2). The resulting progeny from each type of cross among heterozygous individuals were genotyped based on eye-color (the two homozygotes have different eye color –red and white- whereas the heterozygotes are orange-eyed) and examined for different parameters. No statistically significant difference was found for the progeny size and sex ratio among the carriers and non-carriers of the disrupted ultraconserved region (ANOVA, *P*>0.05 in all contrasts) and no deviation from the Mendelian ratios was found either for any heterozygote-by-sex combination analyzed (*G*-test for goodness of fit, *P*>0.05 in all contrasts). See Table S9 for further details on the contrasts performed. Error bars indicate 95% CI.
(TIF)

Figure S10 Relative viability in pairwise competition experiments between embryos of different genotypes at three different starting proportions. (A) The relative viability between two competing genotypes was estimated as (n_1_^′^×n_2_)/(n_1_×n_2_^′^), where n_1_ and n_2_, and n_1_^′^ and n_2_^′^, are the number of embryos and imagoes, respectively. The competing genotypes entail one carrying the tested chromosome (in orange, X_2_: REC, INV1, and INV2) in two possible conditions (heterozygosis, left; homozygosis, right), and the other genotype always in homozygosis (X_1_: SIM1, REV1, and REV2; the tester embryo). The latter always carries the standard arrangement and is invariably *w^−^*. The tested chromosomes differ in whether they carry the ultraconserved region in its intact (REC) or disrupted form (INV1, INV2). (B) Average relative viability between competing genotypes at three different starting proportions. Values were log2 transformed; departures from zero indicate that the competing genotypes differ in their relative viability. The relative viability can be inferred by comparing the different tested chromosomes to the same tester embryo. Eighteen different comparisons were performed: 3 starting proportions×2 genotype conditions for the tested chromosome×3 different tester embryos (Table S10). The starting proportions assayed were 1∶3, 1∶1, and 3∶1 (tester embryo ∶ embryo with the tested chromosome), which are indicated in different colors. For each condition of the tested chromosome and starting proportion, seven combinations of competing genotypes were assayed (2×3×7 = 42 in total). With a few exceptions, the tested chromosomes ranked consistently in their relative viability against a particular tester embryo across starting proportions. Only in one of the experiments (starting proportion, 1∶1; tester embryo, SIM1/SIM1; condition of the tested chromosome, heterozygosis), REC shows significantly higher viability than INV1 and INV2, which denotes a detrimental effect associated with the disruption of the ultraconserved region (Table S10). In the remaining experiments, REC exhibited either an intermediate relative viability in relation to INV1 and INV2 or a relative viability indistinguishable from INV1, INV2, or both. One hundred embryos in total were used per competition setting; every competition setting was replicated 10 times. Error bars indicate 95% CI.
(TIF)

Figure S11 Test for differences in fecundity between males with (INV1, INV2) and without (REC) the disrupted ultraconserved region *CG15121–CG16894*. (A) Progeny size from single-mating experiments between three tester females and the males under scrutiny. The nomenclature of the crosses indicates first the strain of the female and then the strain of the male. No statistically significant differences were found for the crosses examined (Kruskal-Wallis, d.f. = 6; *P*<0.1348; *n* = 7–9). After pooling the data however, INV1, INV2, and REC males are shown to differ in progeny size (Kruskal-Wallis, d.f. = 2; *P*<0.0284; *n* = 16–25), which is due to differences between INV1 and INV2 males (Steel-Dwass; *P*<0.0215; *n* = 16–18; Table S11). (B) Fraction of the progeny sired by red-eyed males in double-mating experiments. For all the crosses, the female genotype is indicated first and the genotypes of the first and second males, which are separated by a comma, are indicated next. Blue, results from SIM1, REV1, and REV2 strains when exposed first to INV1, INV2, or REC males and subsequently to males of their own genotype (direct crosses). Red, results from equivalent experiments in which the order of the males was reversed (reciprocal crosses). A fraction of 0.5 indicates that the sperm of the two males has equivalent fertilization performance. We only considered those days in which progenies sired by the two males were detected. No statistically significant difference was found between INV1, INV2, and REC males irrespective of the order in which they mated (ANOVA; direct crosses: *F*(2,43) = 0.1159, *P*<0.8909, *n* = 10–22; reciprocal crosses: *F*(2,43) = 0.1152, *P*<0.8566, *n* = 14–25; Table S12). Error bars indicate 95% CI.
(TIF)

Figure S12 Average number of fertilized females after exposure to single males with (INV1, INV2) and without (REC) the disrupted ultraconserved region *CG15121–CG16894* during a defined timeframe. Three different timeframes were assayed (1 hr, 3 hr, and 6 hr). Each male was exposed to 10 females of its own strain and 10 males were analyzed per strain and timeframe combination. The exposed females were transferred to individual vials, which were examined for the presence of progeny after 15 days. No differences were found between males with or without the disrupted ultraconserved region *CG15121–CG16894* irrespective of the timeframe assayed (Kruskal-Wallis, d.f. = 2; 1 hr, *P* = 0.1364; 3 hr, *P* = 0.4838; 6 h, *P* = 0.7487; *n* = 10; Table S13). Error bars indicate 95% CI.
(TIF)

Figure S13 Test for differences in global homeostasis among individuals with (INV1, INV2) and without (REC) the disrupted ultraconserved region *CG15121–CG16894* using four proxies. (A) Negative gravitaxis was measured as the average relative height reached in a volumetric cylinder by flies after perturbation. (B) Heat-shock resistance was estimated as the average fraction of flies alive after a heat-shock pulse at 39°C for 30 m. (C) Desiccation resistance was gauged as the average time-to-death of flies under conditions of low humidity. (D) Starvation resistance was assessed as the average time-to-death of flies in the absence to nutrients. The disruption of the ultraconserved region *CG15121–CG16894* has no apparent effect for any of the proxies studied in either sex with the exception of the starvation assay in females (Kruskal-Wallis, d.f. = 2; negative gravitaxis: *P*_males_ = 0.688, *P*_females_ = 0.751, *n* = 20; heat-shock resistance: *P*_males_ = 0.363, *P*_females_ = 0.134, *n* = 5; desiccation resistance: *P*_males_ = 0.964, *P*_females_ = 0.773, *n* = 20; starvation resistance: *P*_males_ = 0.822, *P*_females_<0.0001, *n* = 20; Table S15). For this last sex by proxy combination, the statistically significant differences are associated with the higher resistance of the strain INV2 as compared to INV1 and REC (Steel-Dwass; INV2 vs INV1, *P* = 0.0024; INV2 vs REC, *P* = 0.0002). Lack of differences in the test of negative gravitaxis also discards that the reduced odor attraction of strains INV1 and INV2 to some volatile compounds (Figure 3) could result from a somehow impaired motility. Error bars indicate 95% CI.
(TIF)

Figure S14 Average expression levels for the protein-coding genes encompassed in the ultraconserved region *CG15121–CG16894* across six strains under study. (A) Males; (B) females. Statistically significant differences were assessed using a one-way ANOVA at FDR 0.01. *, statistically significant difference in the general ANOVA (Dataset S1); †, statistically significant difference in at least one of the planned contrasts (Table S17). No consistent differential expression between the strains carrying the disrupted ultraconserved region (INV1, INV2) and the strains with the standard arrangement (REC, SIM1, REV1, REV2) was found. For simplicity, gene order in the standard arrangement for this genomic region is shown. Expression units are arbitrary. Error bars, 95% CI. Note that the confidence interval of the geometric mean is not symmetrical. Green double arrowhead line, inner breakpoint of the inversion *In(2R)51F11-56E2* that disrupts the ultraconserved region. Genes *CG9218*, *CG11025*, *CG30128*, and *CG13873* are represented by several transcripts (Dataset S1).
(PDF)

Figure S15 Average expression levels for eight protein-coding genes flanking the outer breakpoint of the inversion *In(2R)51F11-56E2* across six strains under study. (A) Males; (B) females. Statistically significant differences were assessed using a one-way ANOVA at FDR 0.01 (Dataset S1). No statistically significant differential expression between the strains carrying the disrupted ultraconserved region (INV1, INV2) and the strains without the disruption (REC, SIM1, REV1, REV2) was found for the immediate flanking genes, thus ruling out any artifactual position effect incidentally generated by our procedure. Expression units are arbitrary. Error bars, 95% CI. Note that the confidence interval of the geometric mean is not symmetrical. Green double arrowhead line, outer inversion breakpoint.
(TIF)

Figure S16 Direction of expression change between males and females for the protein-coding genes encompassed in the ultraconserved region *CG15121–CG16894* across six strains. Expression change = 0, no sex bias; expression change>0, overexpression in males; expression change<0, overexpression in females. No consistent differences in the pattern of sex bias in gene expression were found between strains carrying the disrupted ultraconserved region (INV1, INV2) and those carrying the ultraconserved region in its intact form. For simplicity, gene order in the standard arrangement for this genomic region is shown. Statistical significance of the expression change between the sexes was assessed using a one-way ANOVA at FDR 0.01 for each strain separately (Dataset S3). The fold change in expression can be calculated as 2^|direction of expression change|^; the direction of expression change is provided in Dataset S3. Since each strain was analyzed separately, fold change across strains is not comparable. Green double arrowhead line, inner breakpoint disrupting the ultraconserved region *CG15121–CG16894*. Genes *CG9218*, *CG11025*, *CG30128*, and *CG13873* are represented by several transcripts (Dataset S3).
(TIF)

Table S1 Phylogenetic organization of four gene neighborhoods of *D. melanogaster* in other *Drosophila* species according to recent reconstructions of their gene order.
(PDF)

Table S2 Protein-coding genes present in the ultraconserved region *CG15121–CG16894*.
(PDF)

Table S3 Comparative organization of the ultraconserved region *CG15121–CG16894* in *A. gambiae*.
(PDF)

Table S4 Strains.
(PDF)

Table S5 Primers used.
(PDF)

Table S6 Amplicons used to confirm the molecular organization of the genomic regions corresponding to the breakpoints of the inversion *In(2R)51F11-56E2*.
(PDF)

Table S7 Test for differences in progeny size among the strains generated in the course of the experiments using homozygous crosses.
(PDF)

Table S8 Test for differences in sex ratio among the strains generated in the course of the experiments using homozygous crosses.
(PDF)

Table S9 Evaluation of the effect of the disrupted ultraconserved region *CG15121–CG16894* in heterozygosis on a variety of traits.
(PDF)

Table S10 Evaluation of the effect of the disrupted ultraconserved region *CG15121–CG16894* on relative viability in competition experiments prior to imago emergence.
(PDF)

Table S11 Fertility of males carrying the ultraconserved region *CG15121–CG1689* in its disrupted or intact form.
(PDF)

Table S12 Sperm competence of males carrying the ultraconserved region *CG15121–CG1689* in its disrupted or intact form in double mating experiments.
(PDF)

Table S13 Mating ability of flies carrying the ultraconserved region *CG15121–CG1689* in its disrupted or intact form in three different timeframes.
(PDF)

Table S14 Response of flies carrying the ultraconserved region *CG15121–CG1689* in its disrupted or intact form to a variety of volatile compounds.
(PDF)

Table S15 Performance of strains carrying the ultraconserved region *CG15121–CG1689* in its disrupted or intact form based on four proxies of global homeostasis.
(PDF)

Table S16 Expression differences among strains using a one-way ANOVA at FDR 0.01.
(PDF)

Table S17 Expression differences detected in six planned contrasts using one-way ANOVA at FDR 0.01.
(PDF)

Table S18 Statistically significant enrichment for biological coherent patterns (GO term ontology, KEGG pathway, and Interpro domains) among genes differentially expressed in females in at least one of the six planned contrasts.
(PDF)

Table S19 Ability to detect significant differences between flies carrying the ultraconserved region *CG15121–CG1689* in its disrupted or intact form across some of the experiments performed.
(PDF)

Table S20 Number of tRNA, rRNA, and snoRNA genes in the ultraconserved region *CG15121–CG16894* with potential to mediate NAHR events.
(PDF)
