# Supplementary figures and images for: Evaluation of the Role of Functional Constraints on the Integrity of an Ultraconserved Region in the Genus Drosophila
Source: PLoS Genet. 2012 Feb 2;8(2):e1002475. doi: 10.1371/journal.pgen.1002475 (PMC3271063; doi:10.1371/journal.pgen.1002475)

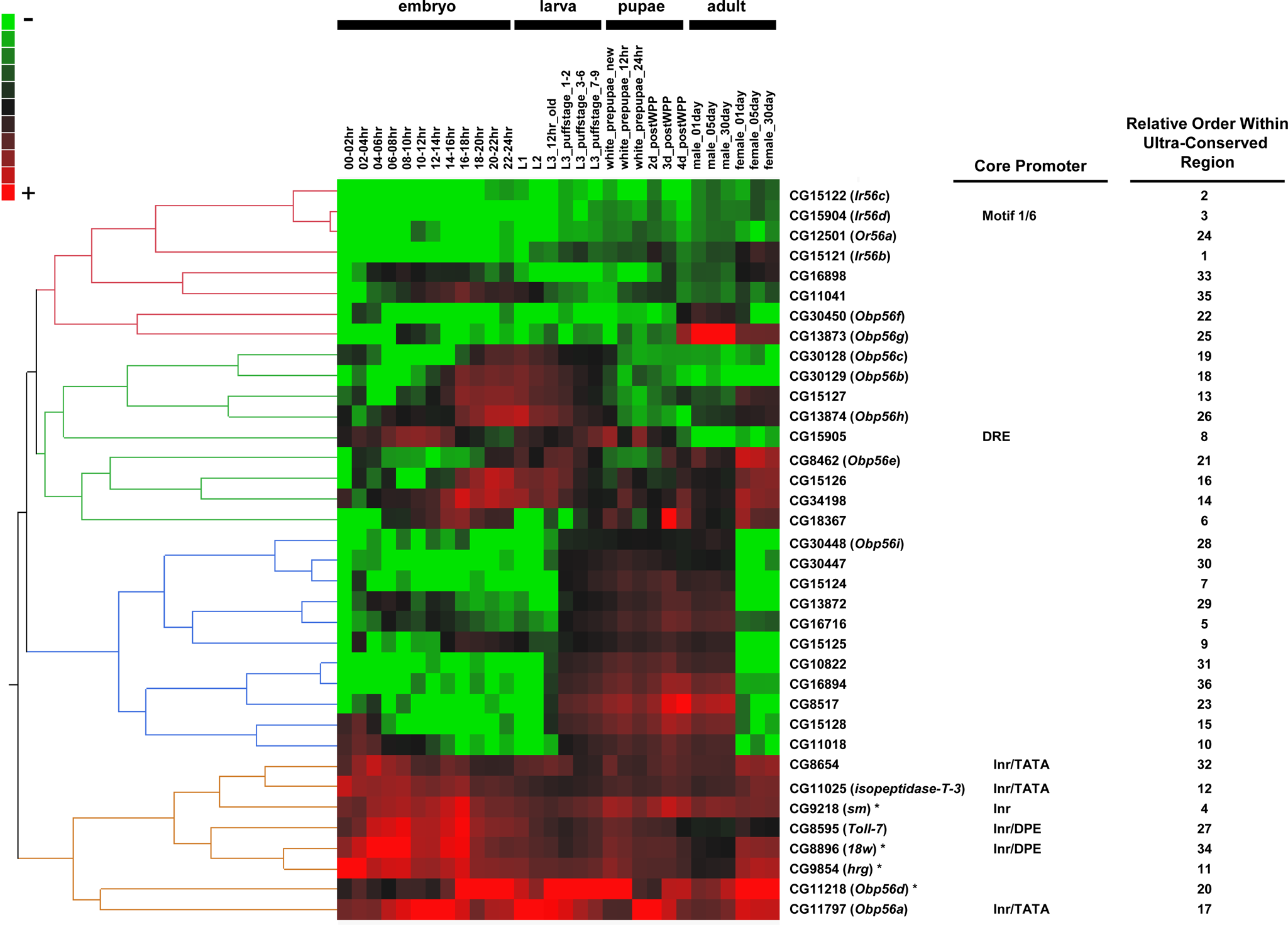

Supplement: Figure S1 — Hierarchical clustering of the expression levels of the protein-coding genes included in the ultraconserved region CG15121–CG16894 during the life cycle of D. melanogaster. Differences in expression levels are color coded (high expression, red ; low expression, green). Asterisks denote genes for which lethal phenotypes have been reported [30], [31]. Predictions for the type of core promoter were obtained using McPromoter [76]. The relative order of the protein-coding genes within the region under study is indicated as in Figure 1 from centromere to telomere. Common names for some genes are indicated in parentheses. (TIF) [file pgen.1002475.s004.tif]

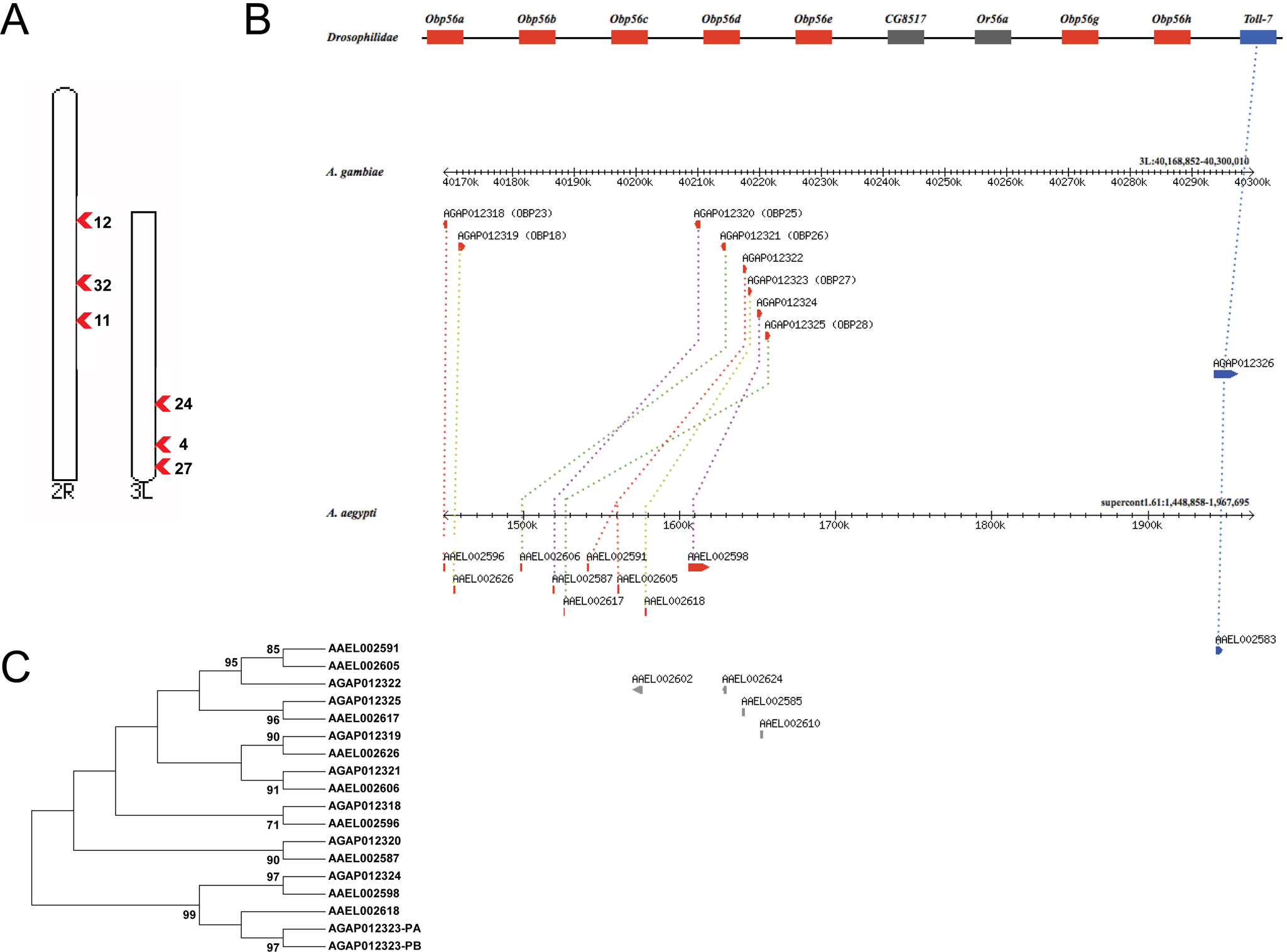

Supplement: Figure S2 — Comparative organization of the ultraconserved region CG15121–CG16894 in Diptera. (A) Chromosomal location (red arrowhead) of six protein-coding genes with reliable one-to-one orthologous relationships between D. melanogaster and A. gambiae [77]. The same numerical code as in Figure 1 is used to indicate the identity of the gene. (B) Conserved collinearity of the gene Toll-7 (blue) and Obp genes (red) across Diptera. Other intervening protein-coding genes are indicated in grey. Drosophila and Anopheles diverged ∼250 mya [83], Anopheles and Aedes diverged ∼150 mya [84], and the divergence time accumulated by the nine Drosophila species previously analyzed was ∼381 my [22]–[24], so that the total divergence time between the Drosophila and mosquito species considered is ∼970 my. Genes Obp56f and Obp56i are not indicated since they are not present in all Drosophila species examined [22], [85]. Doted lines indicate orthologous relationships ([38] and this work). Gene sizes and distances are not to scale in D. melanogaster. (C) Phylogenetic relationships of the OBP protein sequences encoded by genes nearby Toll-7 between A. gambiae and A. aegypti. The percentage of replicate trees in which the associated taxa clustered together in the bootstrap test (1,000 replicates) is shown next to the branches when higher than the cut-off value of 0.5. (TIF) [file pgen.1002475.s005.tif]

A

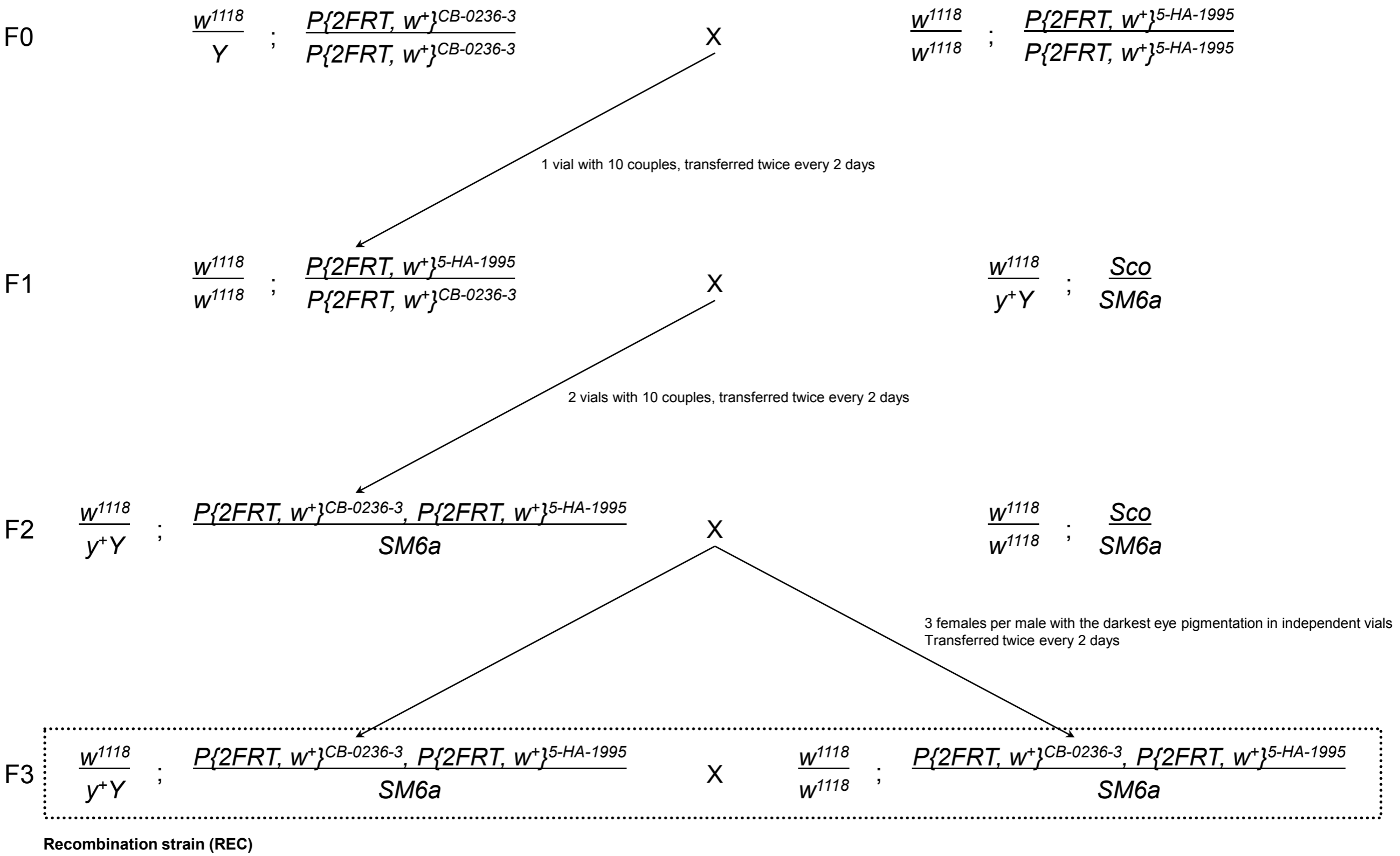

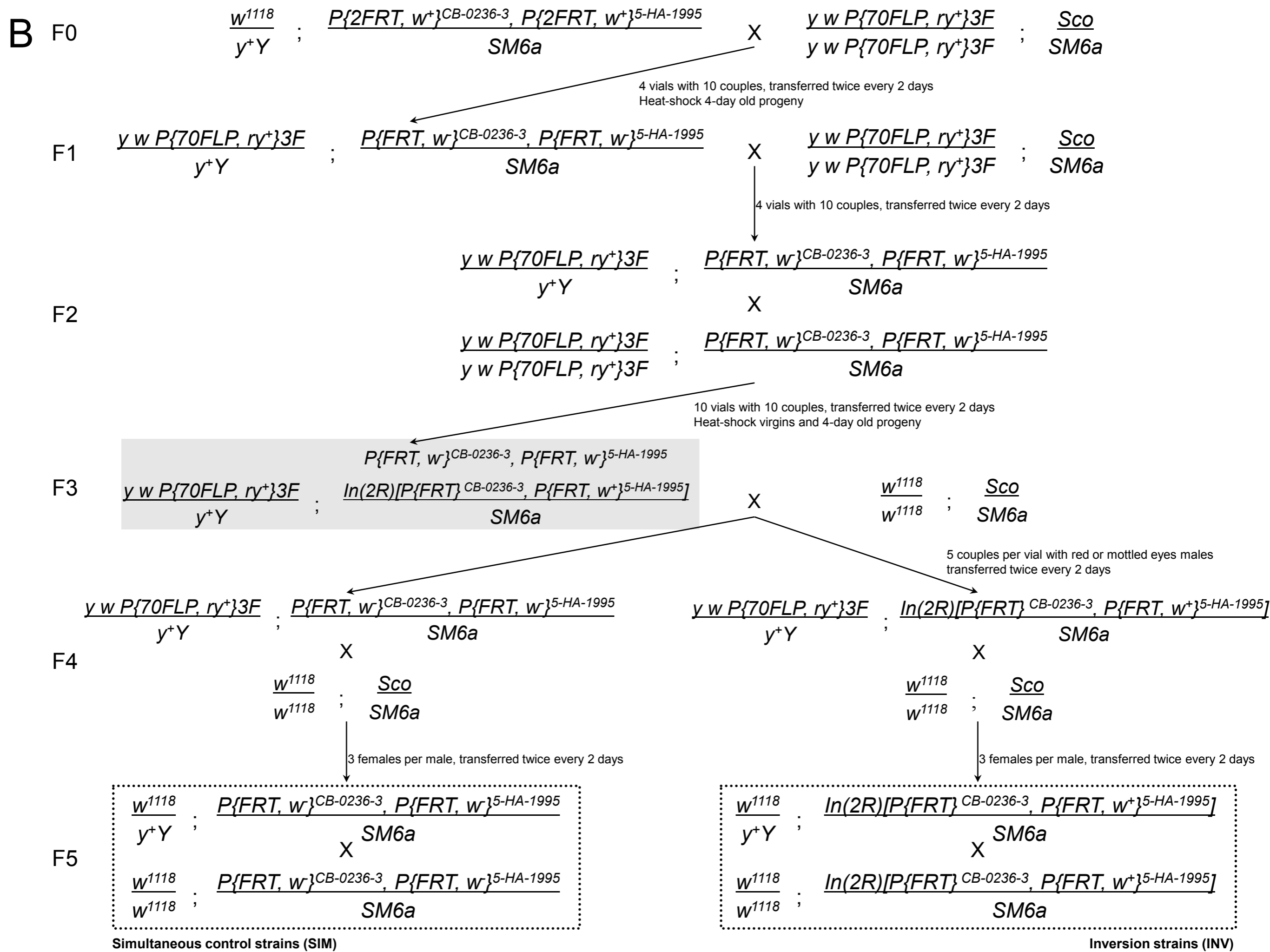

C

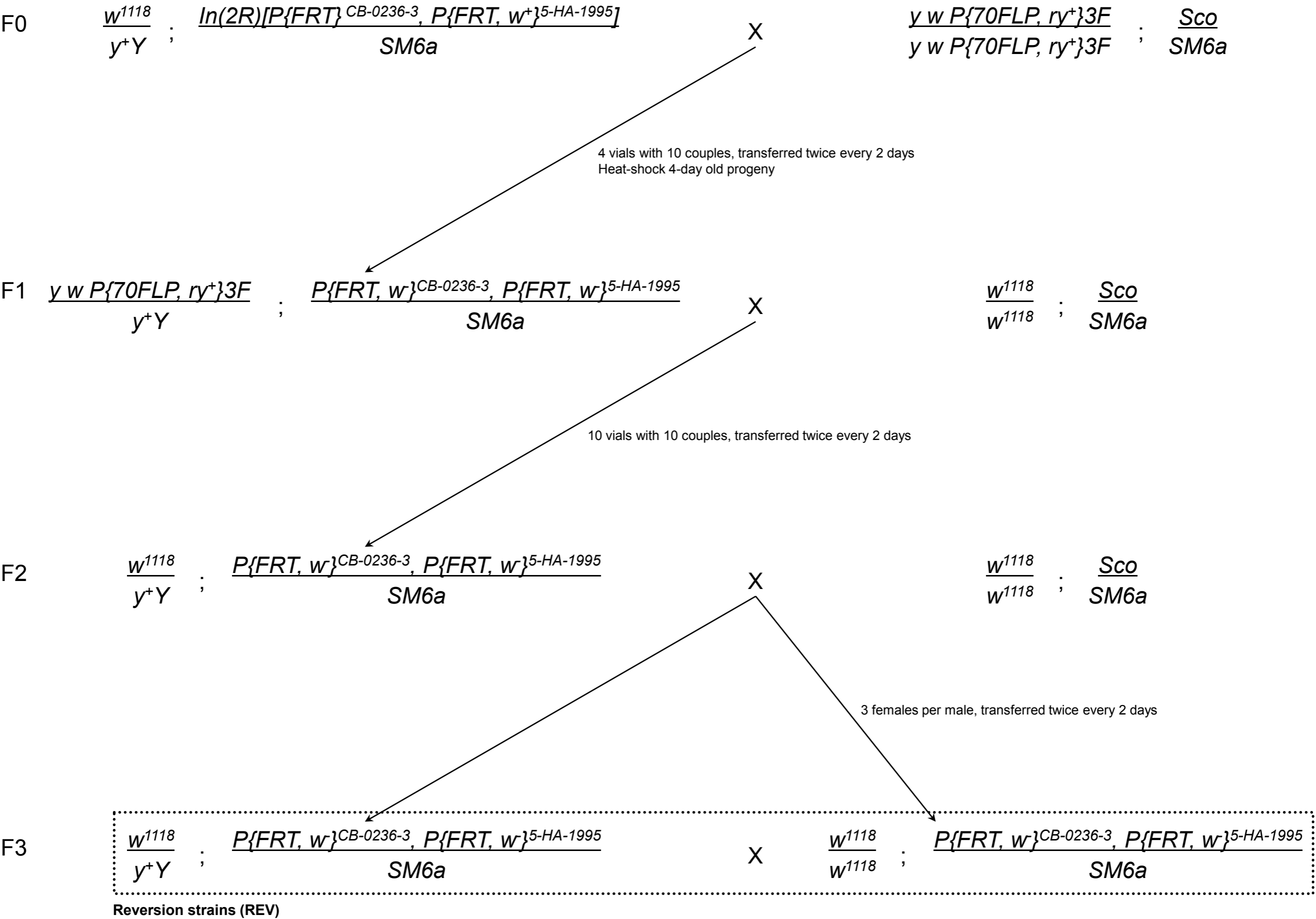

Supplement: Figure S3 — Crossing scheme followed to generate the inversion In(2R)51F11-56E2. (A) Generation of individuals carrying two FRT-bearing elements in cis (REC) upon recombination in the F2. (B) Generation of strains with (INV) or without (SIM for SIMultaneous control) the inversion In(2R)51F11-56E2 using a heat-inducible flippase-recombinase. Notice that both strains derive from progeny generated from the same vial and therefore they have been exposed to the same experimental conditions. Grey box, mosaic flies for the inversion In(2R)51F11-56E2, both in the soma and the germ line. Carriers of the inversion are red-eyed (w+) and therefore readily identifiable. (C) Restoration of standard gene order via a heat-shock inducible reversion (REV for REVertant control). Only relevant chromosomes are indicated for the genotypes. Dotted box, genotype of flies subsequently made homozygous to construct the stocks to be used in further experiments. For clarity, the terminology used here in relation to FRT-bearing TEs and their derivatives in Table S4 is replaced by explicit indications on the number of FRTs and the state of the reporter gene. 2FRT, original TEs (Figures S4 and S5 for further details). (PDF) [file pgen.1002475.s006.pdf]

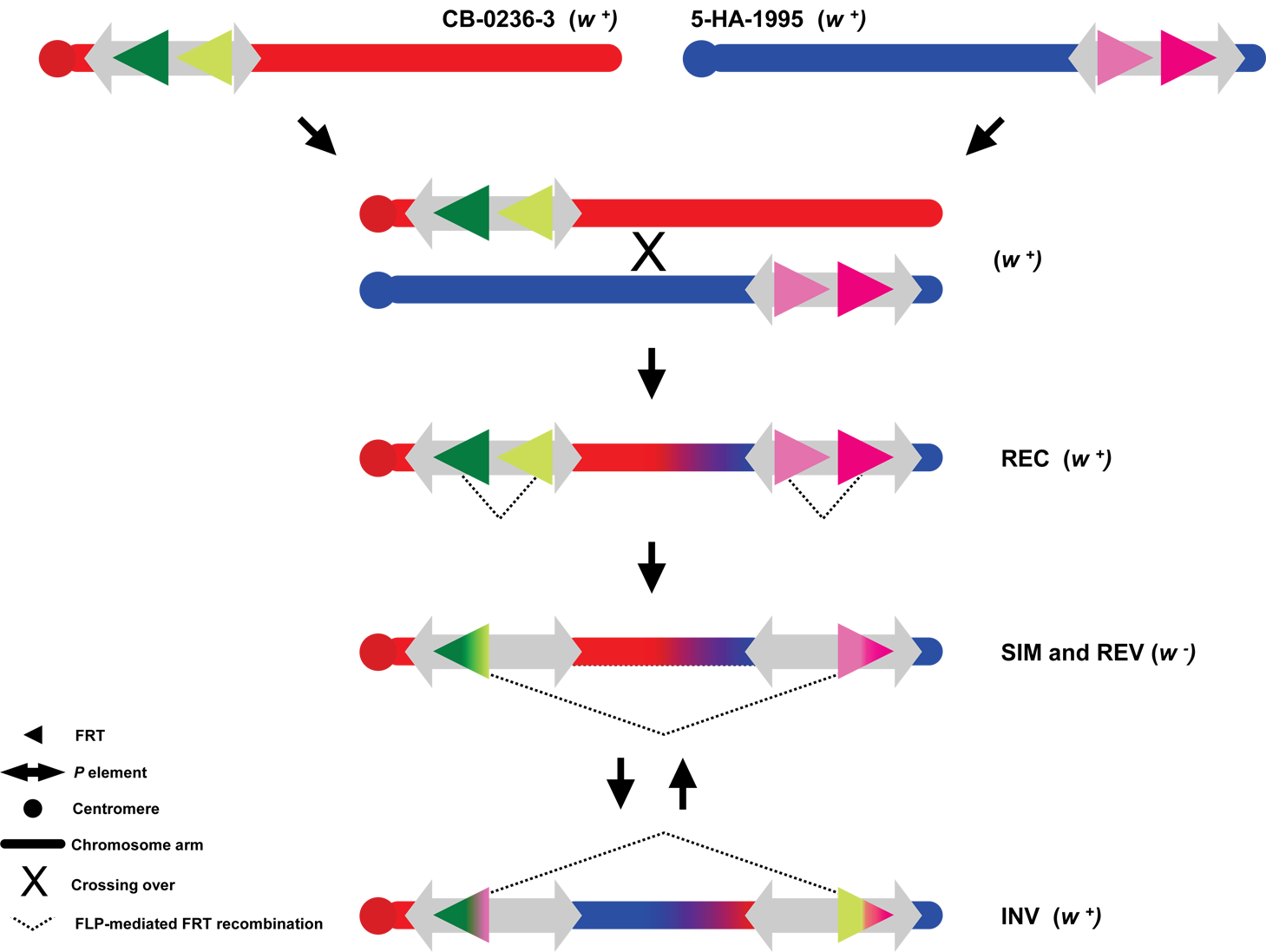

Supplement: Figure S4 — Overview of the chromosomal changes occurred during the generation of the inversion In(2R)51F11-56E2 and the corresponding eye phenotypes. Upon a crossing over event, two starting FRT-bearing TEs are placed on the same homolog. In the presence of a FLP recombinase source, recombination events are heat-shock induced between FRT sequences. The first FLP-mediated recombination event occurs between the two FRT sequences located within each TE leading to two recombined FRT sequences, one at each breakpoint, in opposite orientations. The second recombination event is mediated between these two resulting FRT sequences leading to the generation of the inversion. Phenotypic changes in the eye pigmentation of Drosophila adults are caused by alterations in the reporter gene mini-white carried by the TEs. These alterations occur as a result of the FLP-mediated FRT recombination events. Strains that carry a particular chromosome configuration are indicated. Details on the precise structure of the TEs in each strain are provided in Figure S5. Sizes and distances are not to scale. (TIF) [file pgen.1002475.s007.tif]

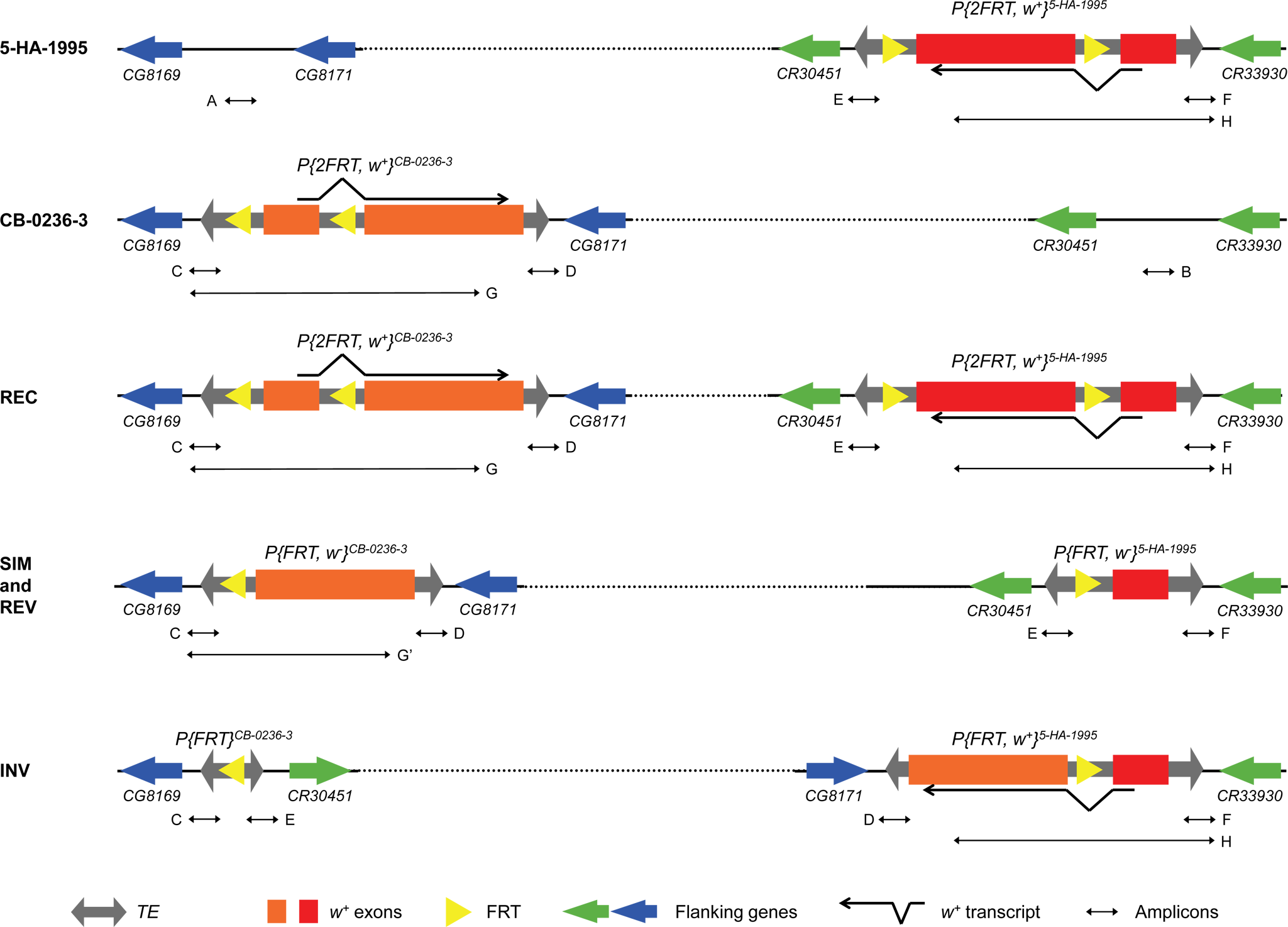

Supplement: Figure S5 — Schematic representation of the molecular configuration of the FRT-bearing TEs at the breakpoint regions of different strains obtained in the course of the generation of the inversion In(2R)51F11-56E2. Two TEs bearing FRT sites in opposite orientation, P{RS3}CB-0236-3 and P{RS5}5-HA-1995, were selected to generate a Type 1 Inversion according to the nomenclature in [42]. Notice that the two FRTs are flanking one of the exons of the modified reporter gene mini-white [86]. While the TEs are intact in the REC strain before the first heat-shock pulse, they undergo different kinds of molecular rearrangements during the rest of the protocol. Specifically, the recombination between the internal FRTs (yellow arrowhead) of each TE leads to the deletion of one of the exons of the reporter gene mini-white (orange and red boxes), which impairs its activity. If the second heat-shock pulse fails to induce a successful NAHR event between the single FRT present in each of the TEs, no rearrangement is generated and no reconstitution of the reporter gene occurs (SIM), which is associated with the w− phenotype. On the contrary, if ectopic recombination occurs, the newly generated inverted arrangement (INV) will be characterized by the presence of a reconstituted reporter gene at one breakpoint (and therefore by the w+ phenotype) and one FRT at the other breakpoint [42]. A subsequent heat-shock pulse can lead to another successful ectopic recombination event restoring the original gene order (REV) and molecular organization at the breakpoint regions as before the inversion. The terminology used in for the FRT-bearing TEs and their derivatives follows that of Figure S4. Amplicons (A–H) used to confirm the molecular configuration of the breakpoint regions in all relevant strains are shown (Tables S5 and S6). Sizes and distances are not to scale. (TIF) [file pgen.1002475.s008.tif]

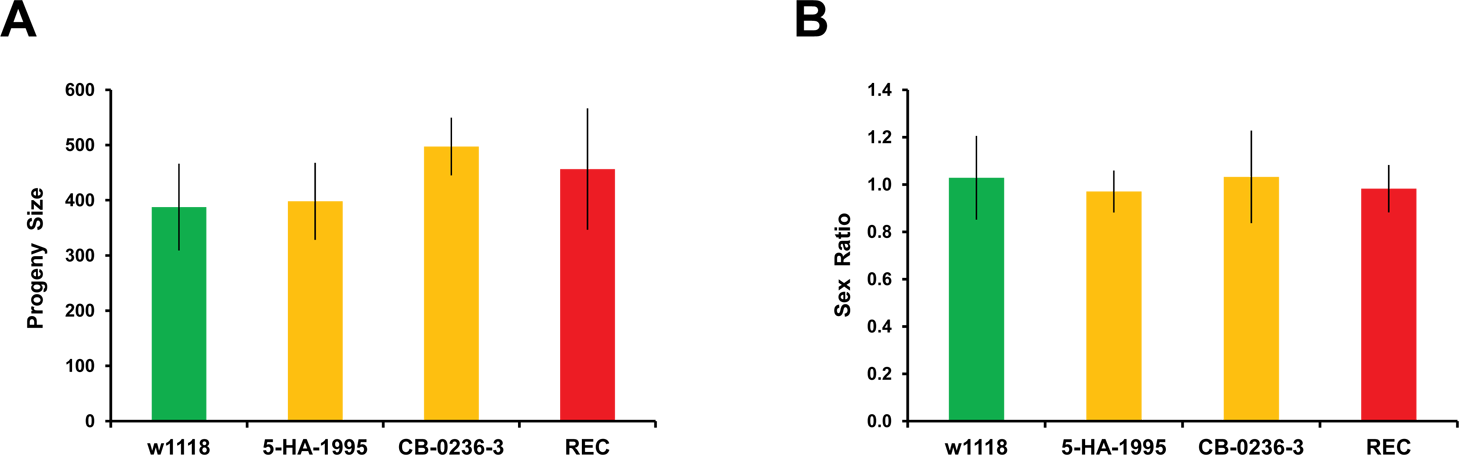

Supplement: Figure S6 — Validation of strains carrying the FRT-bearing TEs used to generate the inversion In(2R)51F11-56E2. (A) Progeny size and (B) sex ratio (female to male) from low-density crosses of homozygous flies for each of the elements alone (5-HA-1995 and CB-0236-3), for both elements in cis (REC), and for flies with the same genetic background but carrying no transposable elements (w1118). No statistically significant difference was found among the strains (Kruskal-Wallis, d.f. = 3; progeny size, P = 0.0730; sex ratio, P = 0.8688; n = 5). Error bars indicate 95% CI. (TIF) [file pgen.1002475.s009.tif]

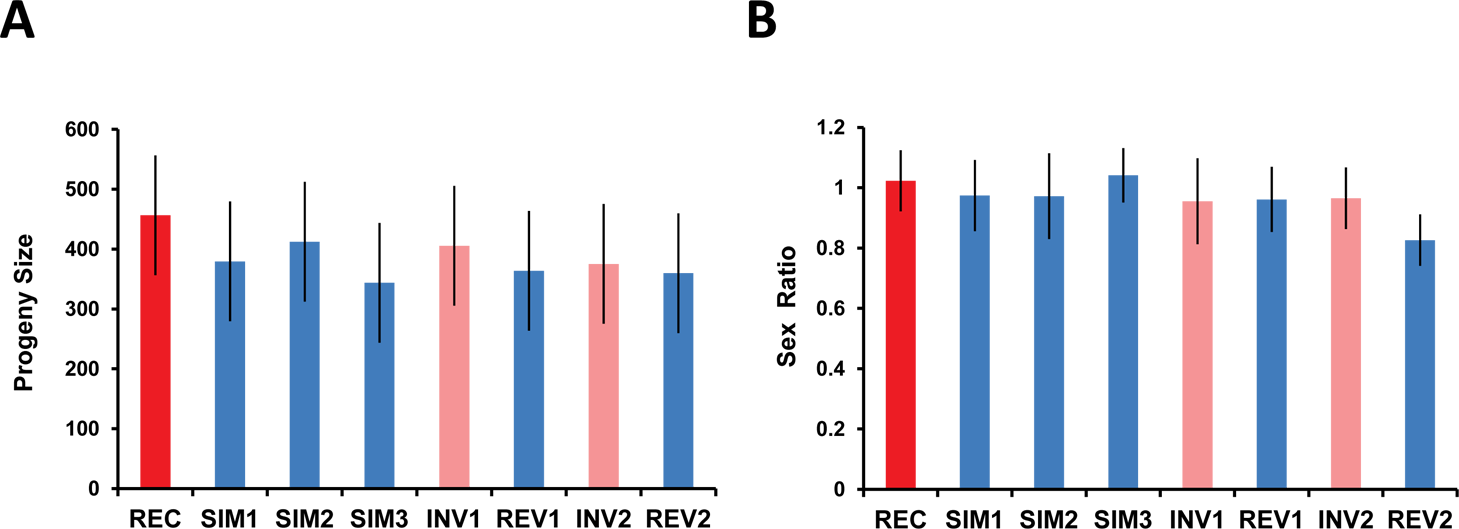

Supplement: Figure S7 — Performance of the strains generated in low-density crosses with homozygous flies. (A) Progeny size and (B) sex ratio (female to male). Kruskal-Wallis test indicated that there are no statistically significant differences among strains both in progeny size (d.f. = 7; P = 0.2308) and in sex ratio (d.f. = 7; P = 0.0863). Pairwise contrasts confirmed that strains carrying the disrupted ultraconserved region CG15121–CG16894 did not show significantly lower values than strains with the ultraconserved region in its intact form (Tables S7 and S8). Error bars indicate 95% CI (n = 5). (TIF) [file pgen.1002475.s010.tif]

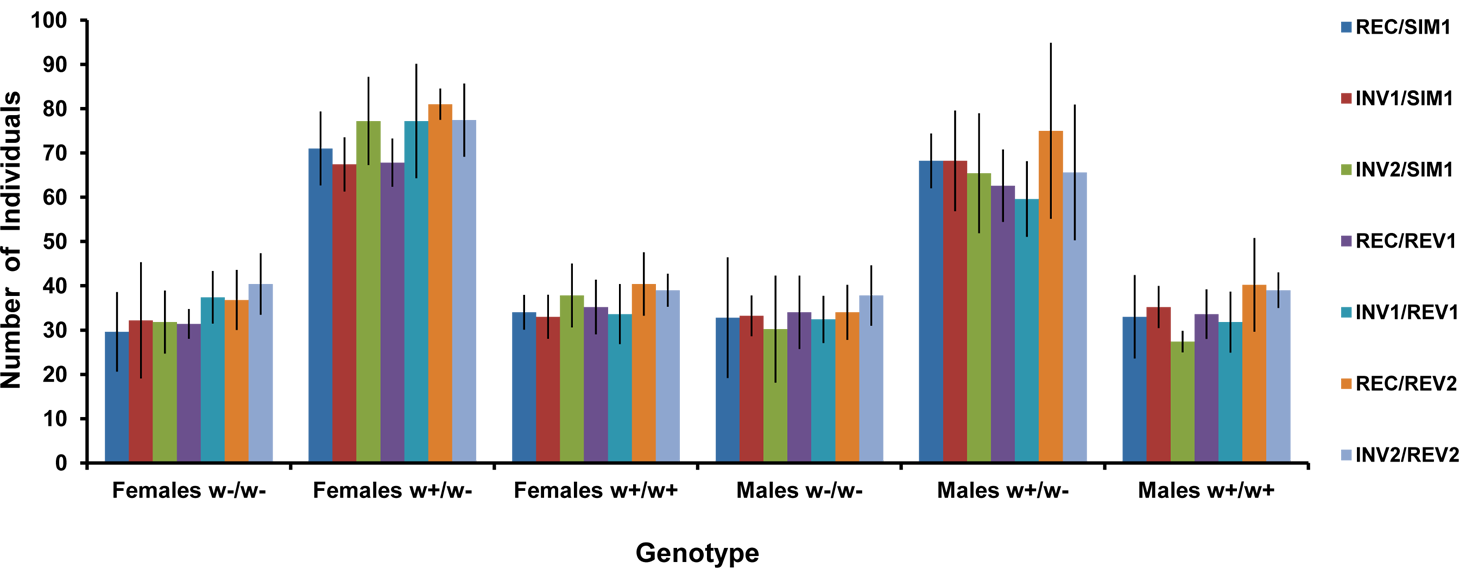

Supplement: Figure S9 — Average progeny size from seven heterozygotes that carry the ultraconserved region CG15121–CG16894 in its disrupted (INV1, INV2) or intact (REC) form. Data for females and males are shown separately. The INV1, INV2, and REC chromosomes were tested in different combination with 2R standard chromosomes associated with the w− phenotype (SIM1, REV1, REV2). The resulting progeny from each type of cross among heterozygous individuals were genotyped based on eye-color (the two homozygotes have different eye color –red and white- whereas the heterozygotes are orange-eyed) and examined for different parameters. No statistically significant difference was found for the progeny size and sex ratio among the carriers and non-carriers of the disrupted ultraconserved region (ANOVA, P>0.05 in all contrasts) and no deviation from the Mendelian ratios was found either for any heterozygote-by-sex combination analyzed (G-test for goodness of fit, P>0.05 in all contrasts). See Table S9 for further details on the contrasts performed. Error bars indicate 95% CI. (TIF) [file pgen.1002475.s012.tif]

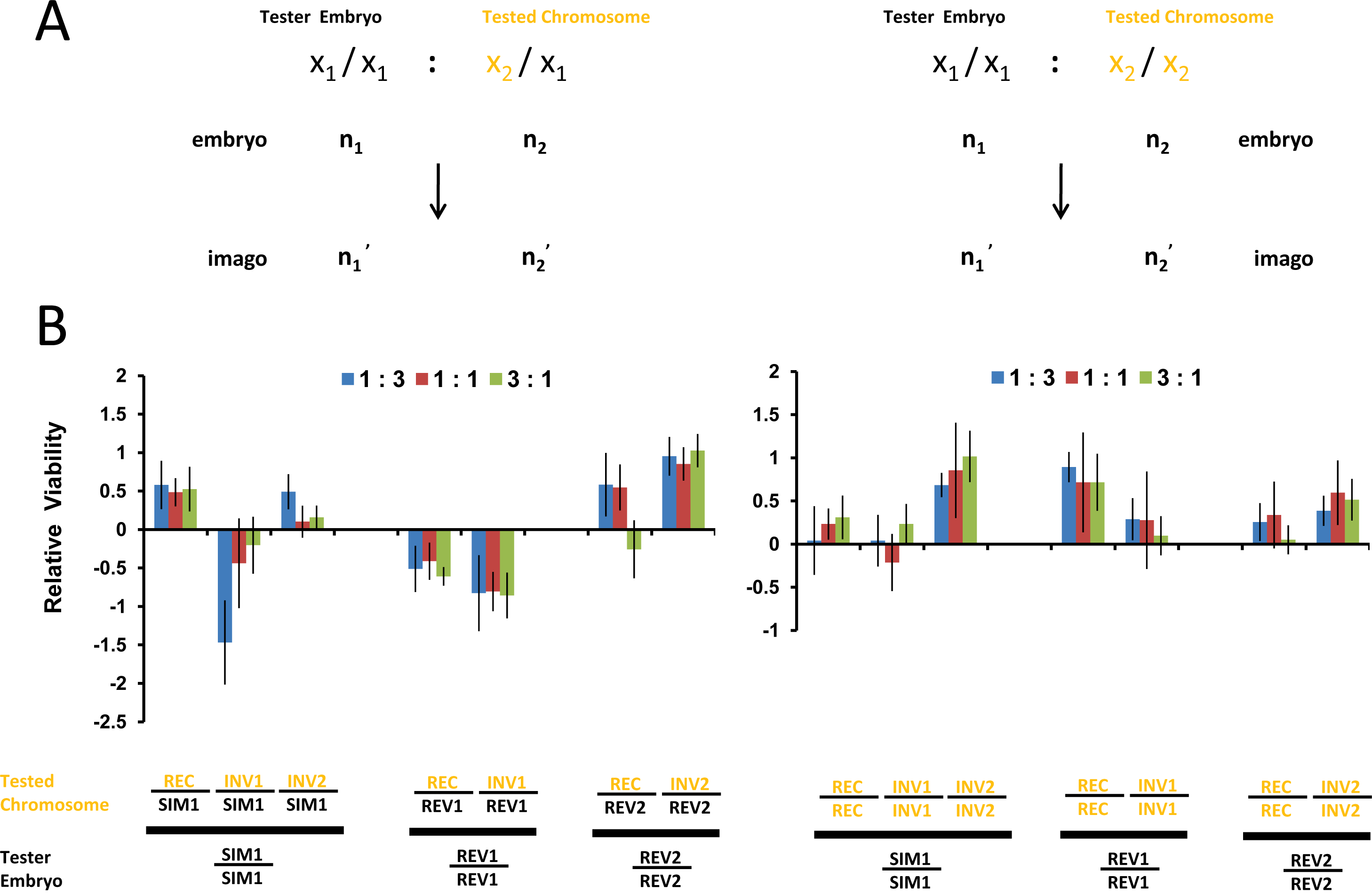

Supplement: Figure S10 — Relative viability in pairwise competition experiments between embryos of different genotypes at three different starting proportions. (A) The relative viability between two competing genotypes was estimated as (n1 ′×n2)/(n1×n2 ′), where n1 and n2, and n1 ′ and n2 ′, are the number of embryos and imagoes, respectively. The competing genotypes entail one carrying the tested chromosome (in orange, X2: REC, INV1, and INV2) in two possible conditions (heterozygosis, left; homozygosis, right), and the other genotype always in homozygosis (X1: SIM1, REV1, and REV2; the tester embryo). The latter always carries the standard arrangement and is invariably w−. The tested chromosomes differ in whether they carry the ultraconserved region in its intact (REC) or disrupted form (INV1, INV2). (B) Average relative viability between competing genotypes at three different starting proportions. Values were log2 transformed; departures from zero indicate that the competing genotypes differ in their relative viability. The relative viability can be inferred by comparing the different tested chromosomes to the same tester embryo. Eighteen different comparisons were performed: 3 starting proportions×2 genotype conditions for the tested chromosome×3 different tester embryos (Table S10). The starting proportions assayed were 1∶3, 1∶1, and 3∶1 (tester embryo ∶ embryo with the tested chromosome), which are indicated in different colors. For each condition of the tested chromosome and starting proportion, seven combinations of competing genotypes were assayed (2×3×7 = 42 in total). With a few exceptions, the tested chromosomes ranked consistently in their relative viability against a particular tester embryo across starting proportions. Only in one of the experiments (starting proportion, 1∶1; tester embryo, SIM1/SIM1; condition of the tested chromosome, heterozygosis), REC shows significantly higher viability than INV1 and INV2, which denotes a detrimental effect associated with the disruptio [file pgen.1002475.s013.tif]

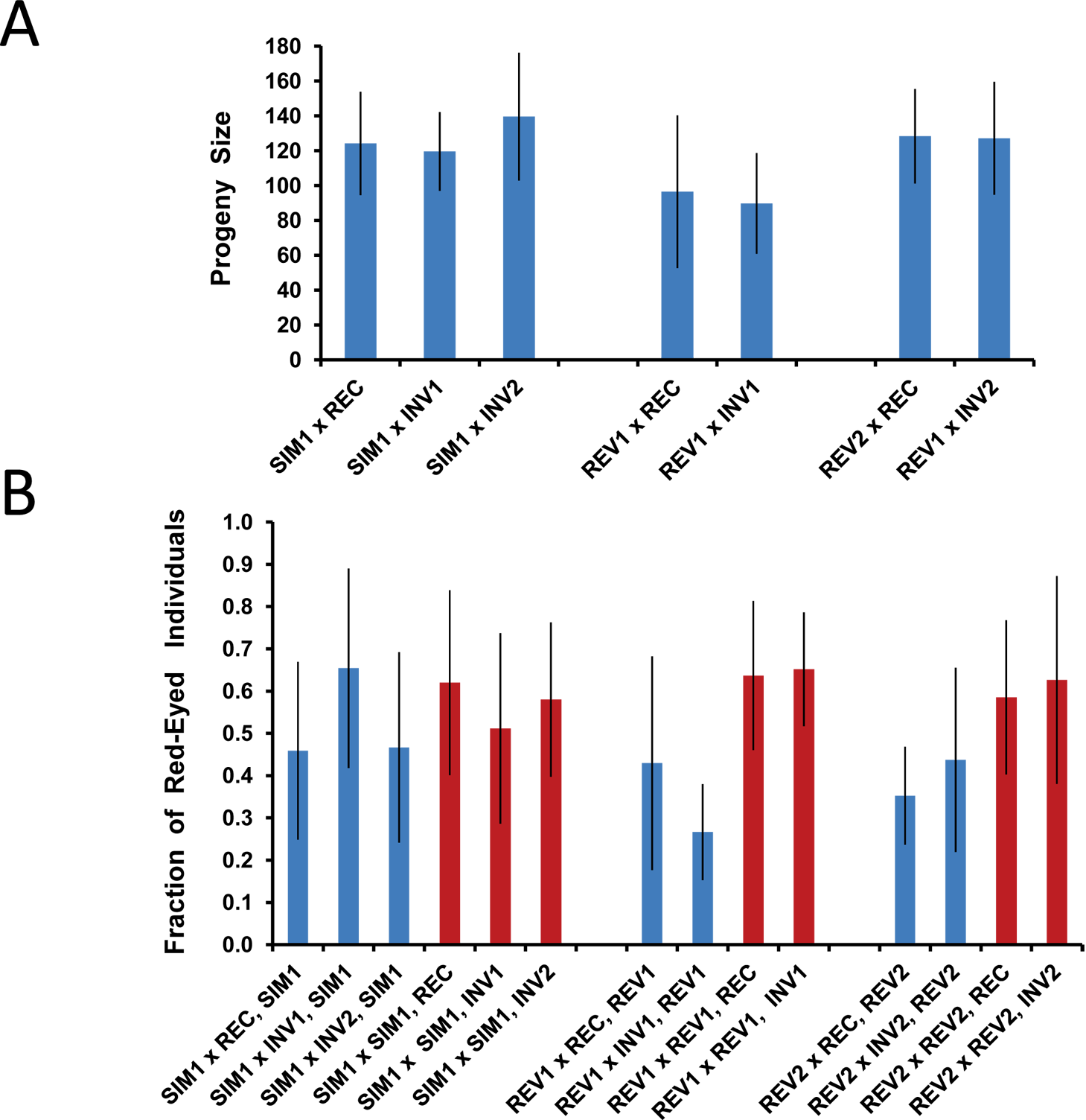

Supplement: Figure S11 — Test for differences in fecundity between males with (INV1, INV2) and without (REC) the disrupted ultraconserved region CG15121–CG16894. (A) Progeny size from single-mating experiments between three tester females and the males under scrutiny. The nomenclature of the crosses indicates first the strain of the female and then the strain of the male. No statistically significant differences were found for the crosses examined (Kruskal-Wallis, d.f. = 6; P<0.1348; n = 7–9). After pooling the data however, INV1, INV2, and REC males are shown to differ in progeny size (Kruskal-Wallis, d.f. = 2; P<0.0284; n = 16–25), which is due to differences between INV1 and INV2 males (Steel-Dwass; P<0.0215; n = 16–18; Table S11). (B) Fraction of the progeny sired by red-eyed males in double-mating experiments. For all the crosses, the female genotype is indicated first and the genotypes of the first and second males, which are separated by a comma, are indicated next. Blue, results from SIM1, REV1, and REV2 strains when exposed first to INV1, INV2, or REC males and subsequently to males of their own genotype (direct crosses). Red, results from equivalent experiments in which the order of the males was reversed (reciprocal crosses). A fraction of 0.5 indicates that the sperm of the two males has equivalent fertilization performance. We only considered those days in which progenies sired by the two males were detected. No statistically significant difference was found between INV1, INV2, and REC males irrespective of the order in which they mated (ANOVA; direct crosses: F(2,43) = 0.1159, P<0.8909, n = 10–22; reciprocal crosses: F(2,43) = 0.1152, P<0.8566, n = 14–25; Table S12). Error bars indicate 95% CI. (TIF) [file pgen.1002475.s014.tif]

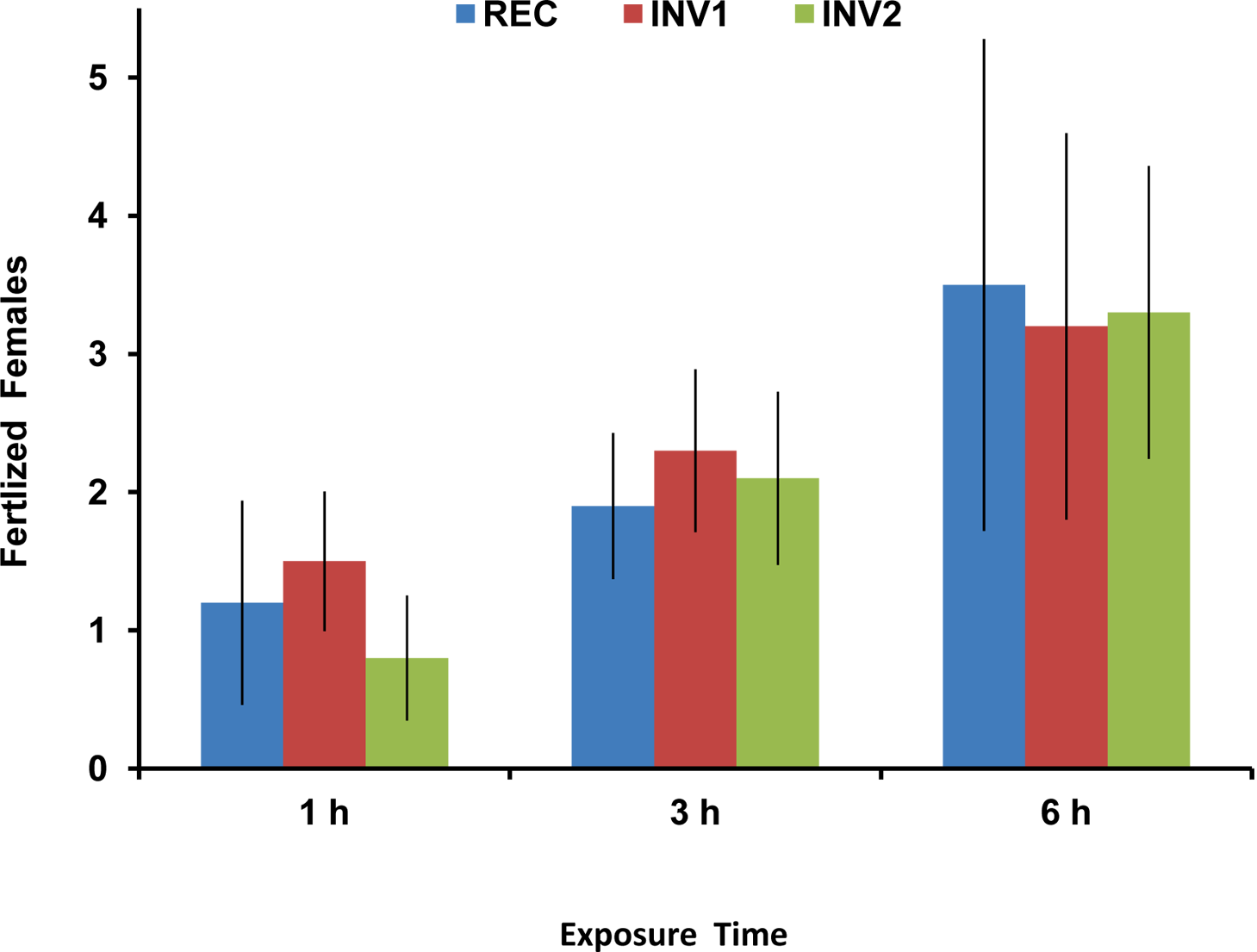

Supplement: Figure S12 — Average number of fertilized females after exposure to single males with (INV1, INV2) and without (REC) the disrupted ultraconserved region CG15121–CG16894 during a defined timeframe. Three different timeframes were assayed (1 hr, 3 hr, and 6 hr). Each male was exposed to 10 females of its own strain and 10 males were analyzed per strain and timeframe combination. The exposed females were transferred to individual vials, which were examined for the presence of progeny after 15 days. No differences were found between males with or without the disrupted ultraconserved region CG15121–CG16894 irrespective of the timeframe assayed (Kruskal-Wallis, d.f. = 2; 1 hr, P = 0.1364; 3 hr, P = 0.4838; 6 h, P = 0.7487; n = 10; Table S13). Error bars indicate 95% CI. (TIF) [file pgen.1002475.s015.tif]

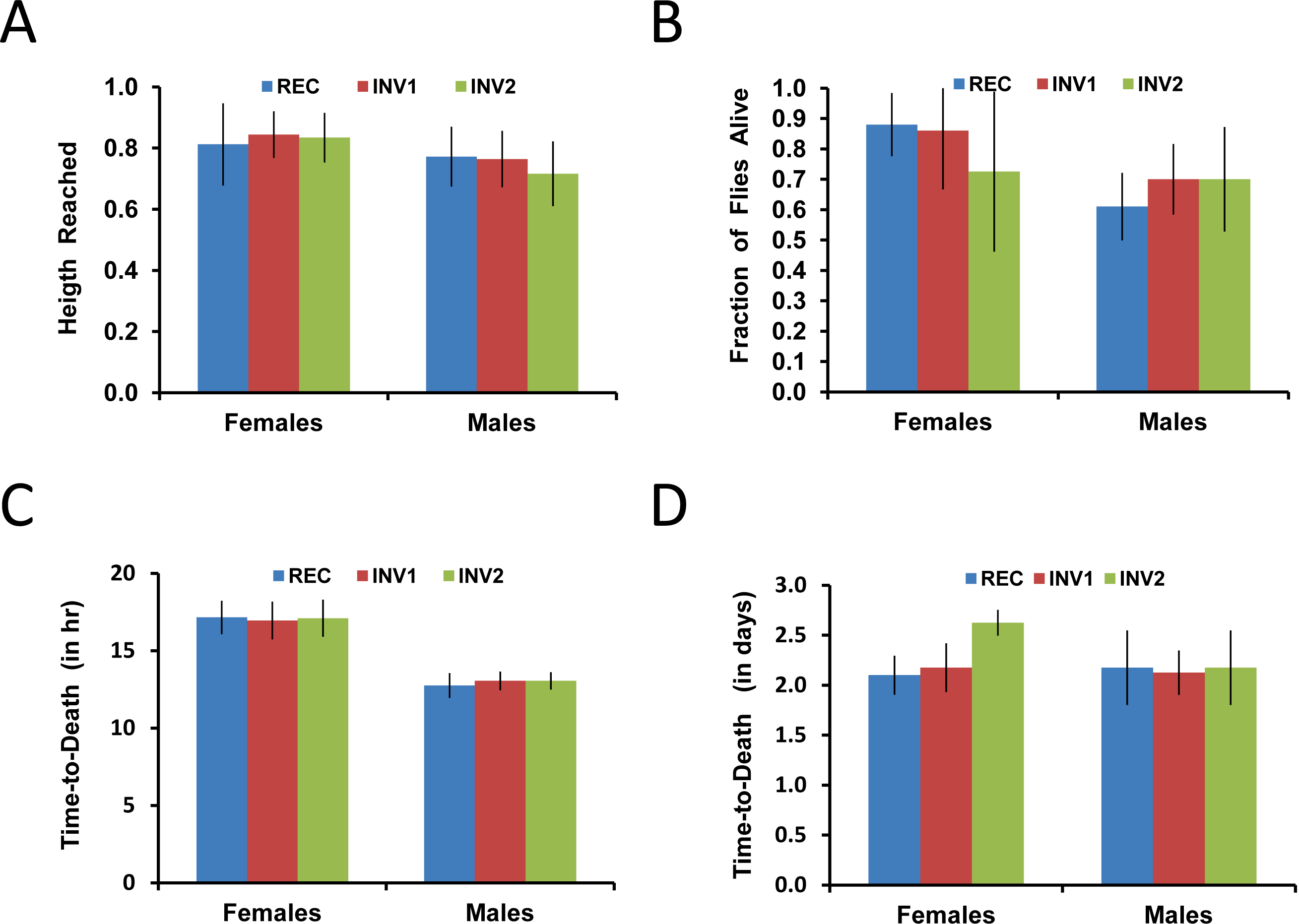

Supplement: Figure S13 — Test for differences in global homeostasis among individuals with (INV1, INV2) and without (REC) the disrupted ultraconserved region CG15121–CG16894 using four proxies. (A) Negative gravitaxis was measured as the average relative height reached in a volumetric cylinder by flies after perturbation. (B) Heat-shock resistance was estimated as the average fraction of flies alive after a heat-shock pulse at 39°C for 30 m. (C) Desiccation resistance was gauged as the average time-to-death of flies under conditions of low humidity. (D) Starvation resistance was assessed as the average time-to-death of flies in the absence to nutrients. The disruption of the ultraconserved region CG15121–CG16894 has no apparent effect for any of the proxies studied in either sex with the exception of the starvation assay in females (Kruskal-Wallis, d.f. = 2; negative gravitaxis: P males = 0.688, P females = 0.751, n = 20; heat-shock resistance: P males = 0.363, P females = 0.134, n = 5; desiccation resistance: P males = 0.964, P females = 0.773, n = 20; starvation resistance: P males = 0.822, P females<0.0001, n = 20; Table S15). For this last sex by proxy combination, the statistically significant differences are associated with the higher resistance of the strain INV2 as compared to INV1 and REC (Steel-Dwass; INV2 vs INV1, P = 0.0024; INV2 vs REC, P = 0.0002). Lack of differences in the test of negative gravitaxis also discards that the reduced odor attraction of strains INV1 and INV2 to some volatile compounds (Figure 3) could result from a somehow impaired motility. Error bars indicate 95% CI. (TIF) [file pgen.1002475.s016.tif]

A

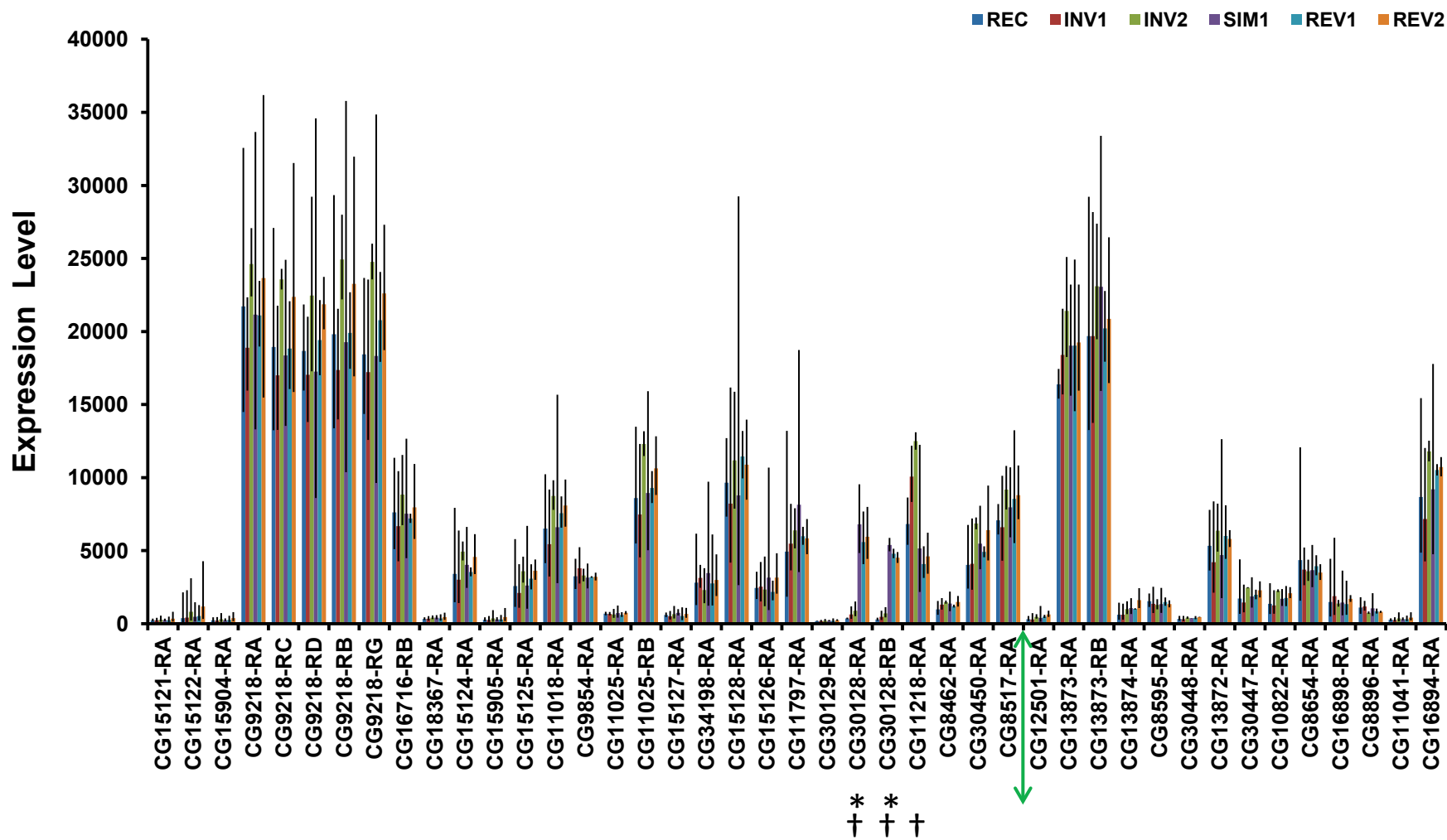

B

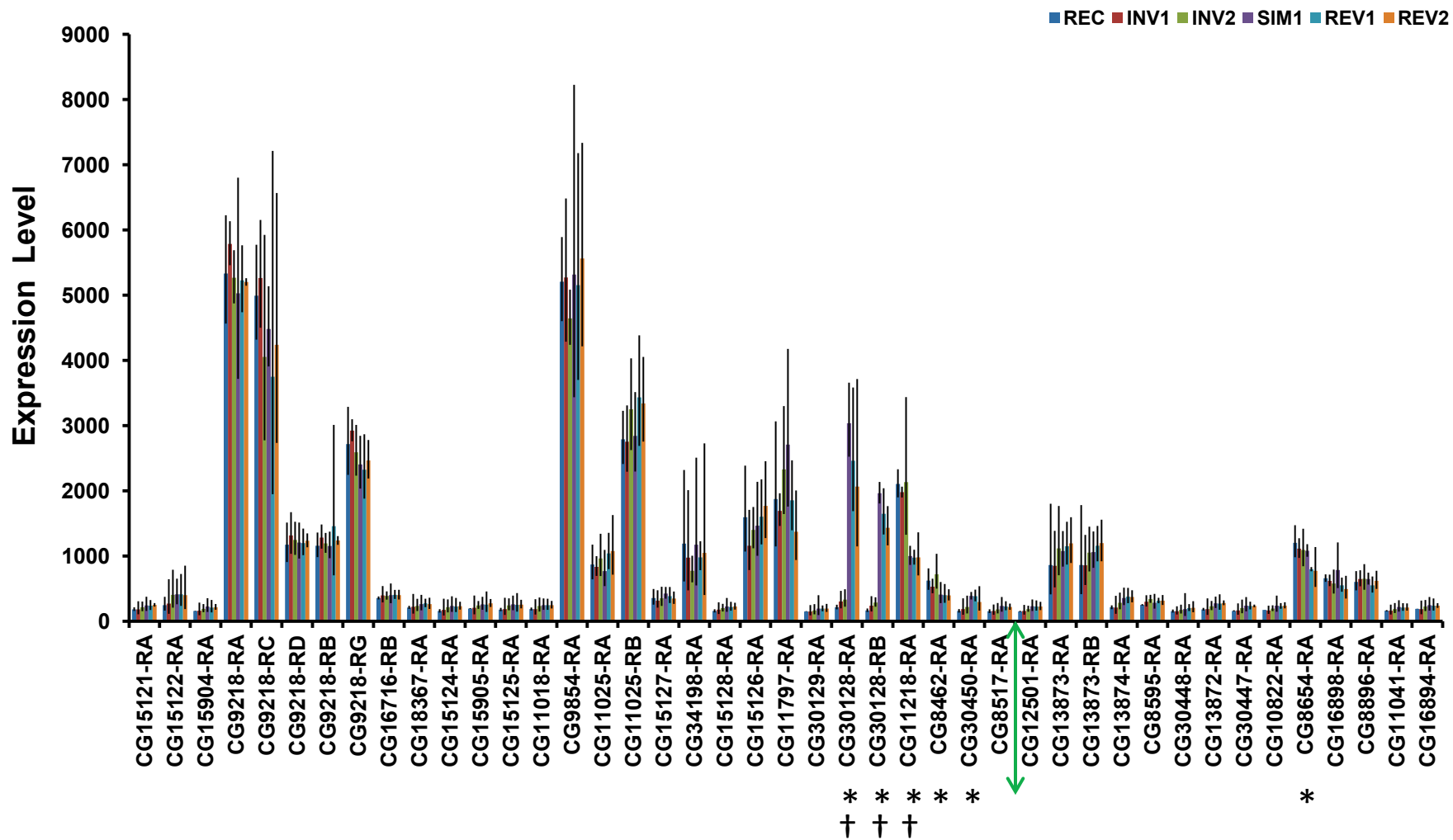

Supplement: Figure S14 — Average expression levels for the protein-coding genes encompassed in the ultraconserved region CG15121–CG16894 across six strains under study. (A) Males; (B) females. Statistically significant differences were assessed using a one-way ANOVA at FDR 0.01. *, statistically significant difference in the general ANOVA (Dataset S1); †, statistically significant difference in at least one of the planned contrasts (Table S17). No consistent differential expression between the strains carrying the disrupted ultraconserved region (INV1, INV2) and the strains with the standard arrangement (REC, SIM1, REV1, REV2) was found. For simplicity, gene order in the standard arrangement for this genomic region is shown. Expression units are arbitrary. Error bars, 95% CI. Note that the confidence interval of the geometric mean is not symmetrical. Green double arrowhead line, inner breakpoint of the inversion In(2R)51F11-56E2 that disrupts the ultraconserved region. Genes CG9218, CG11025, CG30128, and CG13873 are represented by several transcripts (Dataset S1). (PDF) [file pgen.1002475.s017.pdf]

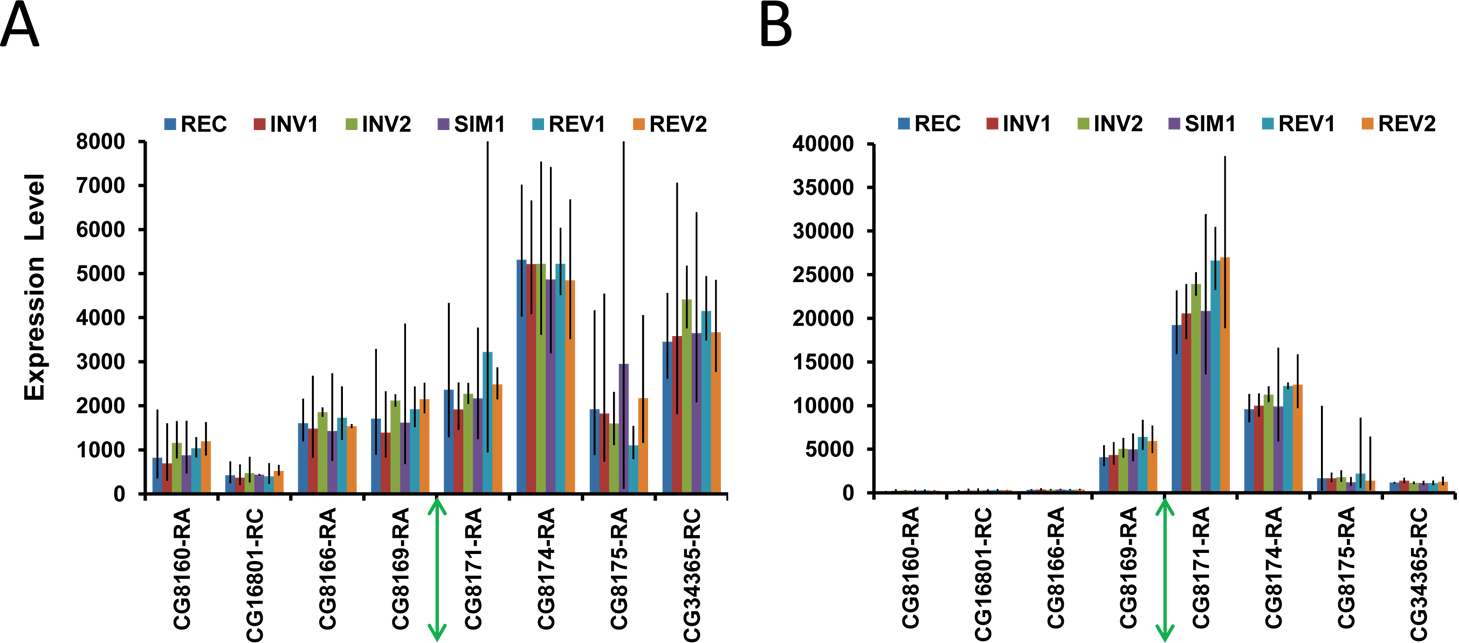

Supplement: Figure S15 — Average expression levels for eight protein-coding genes flanking the outer breakpoint of the inversion In(2R)51F11-56E2 across six strains under study. (A) Males; (B) females. Statistically significant differences were assessed using a one-way ANOVA at FDR 0.01 (Dataset S1). No statistically significant differential expression between the strains carrying the disrupted ultraconserved region (INV1, INV2) and the strains without the disruption (REC, SIM1, REV1, REV2) was found for the immediate flanking genes, thus ruling out any artifactual position effect incidentally generated by our procedure. Expression units are arbitrary. Error bars, 95% CI. Note that the confidence interval of the geometric mean is not symmetrical. Green double arrowhead line, outer inversion breakpoint. (TIF) [file pgen.1002475.s018.tif]

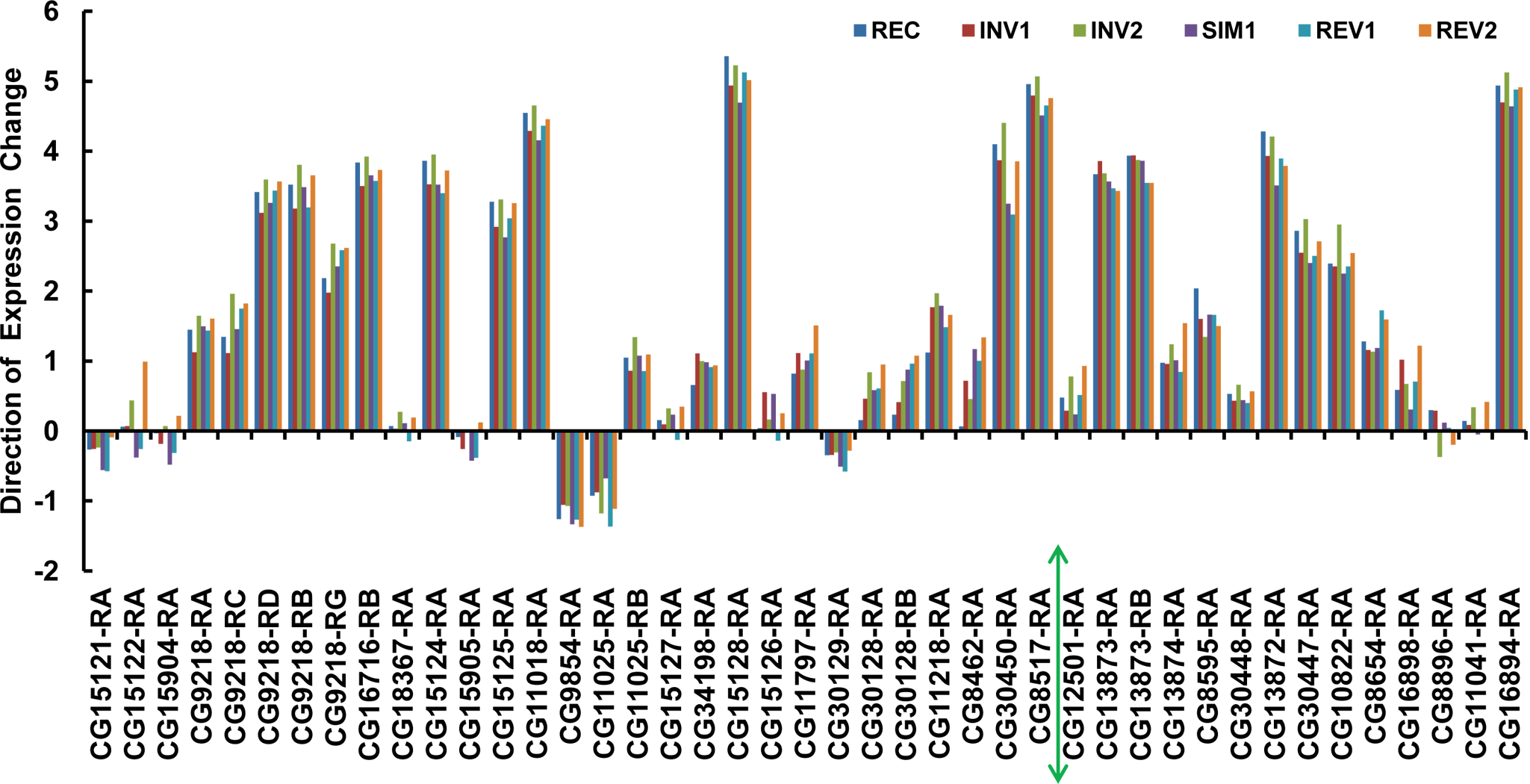

Supplement: Figure S16 — Direction of expression change between males and females for the protein-coding genes encompassed in the ultraconserved region CG15121–CG16894 across six strains. Expression change = 0, no sex bias; expression change>0, overexpression in males; expression change<0, overexpression in females. No consistent differences in the pattern of sex bias in gene expression were found between strains carrying the disrupted ultraconserved region (INV1, INV2) and those carrying the ultraconserved region in its intact form. For simplicity, gene order in the standard arrangement for this genomic region is shown. Statistical significance of the expression change between the sexes was assessed using a one-way ANOVA at FDR 0.01 for each strain separately (Dataset S3). The fold change in expression can be calculated as 2|direction of expression change|; the direction of expression change is provided in Dataset S3. Since each strain was analyzed separately, fold change across strains is not comparable. Green double arrowhead line, inner breakpoint disrupting the ultraconserved region CG15121–CG16894. Genes CG9218, CG11025, CG30128, and CG13873 are represented by several transcripts (Dataset S3). (TIF) [file pgen.1002475.s019.tif]
