# Supplementary material for: Evaluation of the Role of Functional Constraints on the Integrity of an Ultraconserved Region in the Genus Drosophila
Source: PLoS Genet. 2012 Feb 2;8(2):e1002475. doi: 10.1371/journal.pgen.1002475 (PMC3271063; doi:10.1371/journal.pgen.1002475)
Supplement: Figure S8 — Sequence alignment of the region surrounding the FRT-bearing TEs P{RS5}5-HA-1995 and P{RS3}CB-0236, and their derivatives across strains. (A) Outer (2R:11,260,347‥11,261,062) and (B) inner breakpoint (2R:15,613,890‥15,614,461), respectively, of the inversion In(2R)51F11-56E2. No major mutation was incidentally generated during the course of our experiments relative to the strain w1118, which was used by others to generate the strains of the DrosDel collection [41], [42]. The identity of the amplicons sequenced appears in parentheses (Table S6). The direct target sites duplications of the FRT-bearing TEs are easily identified in the region in which the two subsets of sequences overlap within each alignment. Sequence corresponding to TEs is not shown. (PDF) [file pgen.1002475.s011.pdf]

A

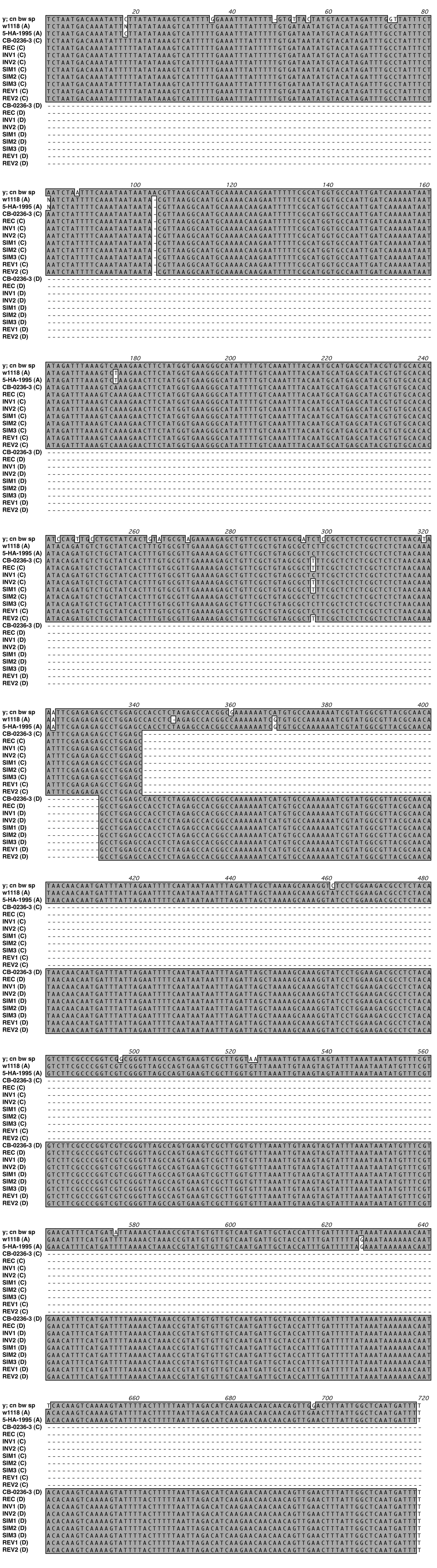

B

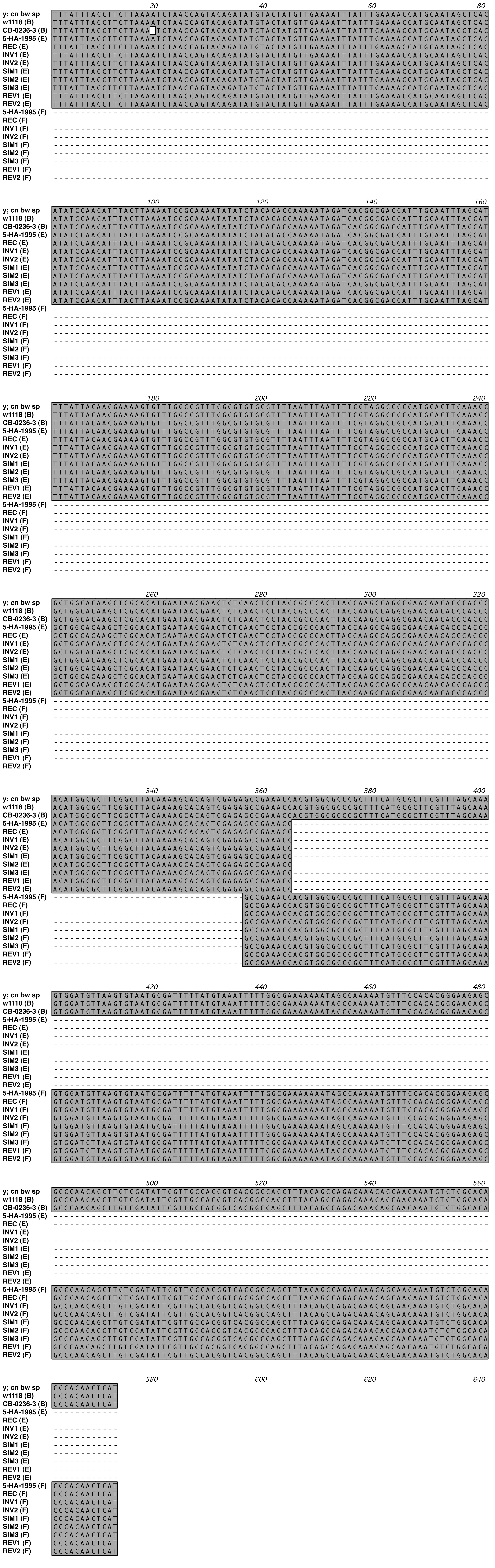
20
40
60
80
100
120
140
160
180
200
220
240
260
280
300
320
340
360
380
400
420
440
460
480
500
520
540
560
580
600
620
640
y; cn bw sp
w1118 (B)
CB-0236-3 (B)
5-HA-1995 (E)
REC (E)
INV1 (E)
INV2 (E)
SIM1 (E)
SIM2 (E)
SIM3 (E)
REV1 (E)
REV2 (E)
5-HA-1995 (F)
REC (F)
INV1 (F)
INV2 (F)
SIM1 (F)
SIM2 (F)
SIM3 (F)
REV1 (F)
REV2 (F)
