# Supplementary material for: Evaluation of the Role of Functional Constraints on the Integrity of an Ultraconserved Region in the Genus Drosophila
Source: PLoS Genet. 2012 Feb 2;8(2):e1002475. doi: 10.1371/journal.pgen.1002475 (PMC3271063; doi:10.1371/journal.pgen.1002475)
Supplement: Table S1 — Phylogenetic organization of four gene neighborhoods of D. melanogaster in other Drosophila species according to recent reconstructions of their gene order. (PDF) [file pgen.1002475.s020.pdf]

Table S1. Phylogenetic organization of four gene neighborhoods of *D. melanogaster* in other *Drosophila* species according to recent reconstructions of their gene order

Neighborhood [a]	Collinear Blocks [b]	Species Where Disrupted [c]
CG15446-CG1494	553, 554, 555	*wil, pse*
CG4767-CG17329	892, 893, 894, 895, 897, 898, 899	*ana, pse, wil, vir, moj, gri*
CG13330-CG13440	1208, 1209, 1210, 1211, 1212	*ere, yak, pse, wil, vir, moj, gri*
CG14225-CG33931	527, 528, 529, 530, 531, 532	*yak, ana, pse, wil, vir, moj, gri*

Based on comparative data from [1].

[a] Empirically disrupted in *D. melanogaster* [2].

[b] Numerical code of those collinear blocks in which the genes of the neighborhood are located. Collinear blocks were delineated based on conservation of gene order, but not necessarily on conservation of gene orientation (GO definition), across nine *Drosophila* species; gene transpositions in and out were allowed [1].

[c] *ere, D. erecta; yak, D. yakuba; ana, D. ananassae; pse, D. pseudoobscura; wil, D. willistoni; vir, D. virilis; moj, D. mojavensis; gri, D. grimshawi.*

Supporting References

1. von Grotthuss M, Ashburner M, Ranz JM (2010) Fragile regions and not functional constraints predominate in shaping gene organization in the genus Drosophila. Genome Res 20: 1084-1096.

2. Meadows LA, Chan YS, Roote J, Russell S (2010) Neighbourhood continuity is not required for correct testis gene expression in Drosophila. PLoS Biol 8: e1000552.
