# Supplementary material for: Evaluation of the Role of Functional Constraints on the Integrity of an Ultraconserved Region in the Genus Drosophila
Source: PLoS Genet. 2012 Feb 2;8(2):e1002475. doi: 10.1371/journal.pgen.1002475 (PMC3271063; doi:10.1371/journal.pgen.1002475)
Supplement: Table S2 — Protein-coding genes present in the ultraconserved region CG15121–CG16894. (PDF) [file pgen.1002475.s021.pdf]

Table S2. Protein-coding genes present in the ultraconserved region *CG15121-CG16894*

Code [a]	Gene [b]	Cross-Mapped [c]	FlyAtlas [d] Expression	FlyAtlas [d] Enrichment	SEBIDA Classification [e]	Male-Biased Gene Expression [f]	Sperm Proteome [g]	Seminal Fluid Proteome [h]	Chemosensory Perception [i]
1	*CG15121*	✓			Unbiased (female)				
2	*CG15122*	✓			Unclassified				
3	*CG15904*	✓			Female				
4	*CG9218*	✓	bhcmzotaxywgs	t	Male	mel, sim, yak, ana, pse, vir, moj			
5	*CG16716*	✓	tay	t	Male	mel, sim, yak, ana, pse, vir, moj			
6	*CG18367*	✓	bmwg		Male				
7	*CG15124*	✓	ty	t	Male	mel, sim, yak, ana, pse, vir, moj			
8	*CG15905*	✓			Unbiased (female)				
9	*CG15125*	✓	t	t	Male	mel, sim, yak, ana, pse, vir, moj			
10	*CG11018*		ty	t	Male		✓		
11	*CG9854*	✓	bhcmzotaxywgs		Female				
12	*CG11025*	✓	bhcmzotaxywgs		Male	mel, sim, yak, pse, vir			
13	*CG15127*	✓	m	m	Female				
14	*CG34198*				NA				
15	*CG15128*	✓	ty	t	Male	mel, sim, yak, ana, pse, moj	✓		
16	*CG15126*	✓			Unclassified				
17	*CG11797*	✓	hztw		Unbiased (male)				*Obp56a*
18	*CG30129*	✓			Female				*Obp56b*
19	*CG30128*	✓			Unclassified				*Obp56c*

Table S2. Protein-coding genes present in the ultraconserved region *CG15121-CG16894*

		Cross-	FlyAtlas [d]		SEBIDA	Male-Biased	Sperm	Seminal Fluid	Chemosensory
Code [a]	Gene [b]	Mapped [c]	Expression	Enrichment	Classification [e]	Gene Expression [f]	Proteome [g]	Proteome [h]	Perception [i]
20	*CG11218*	✓	bhcztxwg		Male	mel, sim, yak			*Obp56d*
21	*CG8462*	✓	hczaw		Female			mel, sim, yak	*Obp56e*
22	*CG30450*		a	a	Male			mel, sim, yak	*Obp56f*
23	*CG8517*	✓	tay	t	Male	mel, sim, yak, ana, pse, moj			
24	*CG12501*	✓			Unclassified				*Or56a*
25	*CG13873*	✓	haw		Male	mel, sim, yak, ana, pse		mel, sim, yak	*Obp56g*
26	*CG13874*	✓	h	h	Unbiased (male)	sim, yak, vir, moj			*Obp56h*
27	*CG8595*	✓			Male				
28	*CG30448*		ta	a	Male			mel, sim, yak	*Obp56i*
29	*CG13872*	✓	t	t	Male	yak, ana, pse, vir, moj			
30	*CG30447*		ty	t	Male				
31	*CG10822*		ty	t	Male				
32	*CG8654*	✓	hcztxywgs		Male	ana, pse, moj			
33	*CG16898*		hmztxw	m	Male				
34	*CG8896*	✓	bhcmzotaxywgs		Unbiased (female)				
35	*CG11041*	✓			Unbiased (female)				
36	*CG16894*	✓	ty	t	Male	mel, sim, yak, ana, pse, vir, moj			

[a] As in Figure 1.

[b] FlyBase [1].

[c] Inferred to be present within this ultraconserved region prior to the divergence of the lineages that lead to the *Sophophora* and *Drosophila* subgenera [2].

[d] Tissues with evidence of expression [3] (brain, b; carcass, w; crop, c; head, h; hindgut, z; larval fat body, y; larval Malphigian tubule, x; male accessory gland, a; midgut, m; ovary, o; salivary gland, s; testis, t; and thoracic abdominal ganglion, g) and preferential expression (five-fold increase in relation to the whole body) as estimated previously [2].

[e] Consensus categorization of the sex bias in mRNA abundance based on a reanalysis of several expression datasets [4]. The trend is indicated in parentheses for those cases in which no statistically significant bias was found.

[f] As categorized based on species-specific microarray experiments in *D. melanogaster -mel-*, *D. simulans -sim-*, *D. yakuba -yak-*, *D. ananassae -ana-*, *D. pseudoobscura -pse-*, *D. virilis -vir-*, and *D. mojavensis -moj-* [5].

[g] As assessed by whole-sperm mass spectroscopy in *D. melanogaster* [6].

[h] Species in which evidence of expression was found by mass spectroscopy in recently mated females [7].

[i] Common name as a member of a multigene family. Those encoding odorant-binding proteins are organized into two clusters: *Obp56a-Obp56f* and *Obp56g-Obp56i* [8].

Supporting References

1. Tweedie S, Ashburner M, Falls K, Leyland P, McQuilton P, et al. (2009) FlyBase: enhancing Drosophila Gene Ontology annotations. Nucleic Acids Res 37: D555-559.

2. von Grotthuss M, Ashburner M, Ranz JM (2010) Fragile regions and not functional constraints predominate in shaping gene organization in the genus Drosophila. Genome Res 20: 1084-1096.

3. Chintapalli VR, Wang J, Dow JA (2007) Using FlyAtlas to identify better Drosophila melanogaster models of human disease. Nat Genet 39: 715-720.

4. Gnad F, Parsch J (2006) Sebida: a database for the functional and evolutionary analysis of genes with sex-biased expression. Bioinformatics 22: 2577-2579.

5. Zhang Y, Sturgill D, Parisi M, Kumar S, Oliver B (2007) Constraint and turnover in sex-biased gene expression in the genus Drosophila. Nature 450: 233-237.

6. Dorus S, Busby SA, Gerike U, Shabanowitz J, Hunt DF, et al. (2006) Genomic and functional evolution of the Drosophila melanogaster sperm proteome. Nat Genet 38: 1440-1445.

7. Findlay GD, Yi X, Maccoss MJ, Swanson WJ (2008) Proteomics reveals novel Drosophila seminal fluid proteins transferred at mating. PLoS Biol 6: e178.

8. Vieira FG, Sanchez-Gracia A, Rozas J (2007) Comparative genomic analysis of the odorant-binding protein family in 12 Drosophila genomes: purifying selection and birth-and-death evolution. Genome Biol 8: R235.
