# Supplementary material for: Evaluation of the Role of Functional Constraints on the Integrity of an Ultraconserved Region in the Genus Drosophila
Source: PLoS Genet. 2012 Feb 2;8(2):e1002475. doi: 10.1371/journal.pgen.1002475 (PMC3271063; doi:10.1371/journal.pgen.1002475)
Supplement: Table S3 — Comparative organization of the ultraconserved region CG15121–CG16894 in A. gambiae. (PDF) [file pgen.1002475.s022.pdf]

Table S3. Comparative organization of the ultraconserved region *CG15121-CG16894* in *A. gambiae*

Protein-Coding Gene [a]	Chromosome	Ensembl Gene ID	Coordinates (AgampP3)
CG9218	3L	AGAP012054	36975510..36983150
CG9854	2R	AGAP013207	36911284..36914633
CG11025	2R	AGAP002436	21377985..21379909
CG12501	3L	AGAP011631	30452735..30454242
CG8595	3L	AGAP012326	40293607..40297410
CG8654	2R	AGAP003039	31351382..31364077

[a] Orthologues in *D. melanogaster* for which a one-to-one relationship exists [1].

Supporting References

1. Haider S, Ballester B, Smedley D, Zhang J, Rice P, et al. (2009) BioMart Central Portal--unified access to biological data. Nucleic Acids Res 37: W23-27.
