# Supplementary material for: Evaluation of the Role of Functional Constraints on the Integrity of an Ultraconserved Region in the Genus Drosophila
Source: PLoS Genet. 2012 Feb 2;8(2):e1002475. doi: 10.1371/journal.pgen.1002475 (PMC3271063; doi:10.1371/journal.pgen.1002475)
Supplement: Table S4 — Strains. (PDF) [file pgen.1002475.s023.pdf]

Table S4. Strains

Genotype	Abbreviated Name	Cytological Description
w1118; 2iso; 3iso [a]	w^{1118}	2Rc—2Rt
w1118; P{RS5}5-HA-1995; 3iso [b]	5-HA-1995	2Rc—2Rt
w1118; P(RS3}CB-0236-3; 3iso [b]	CB-0236-3	2Rc—2Rt
w1118; Sco/SM6; 3iso [a]	-	2Rc—2Rt
y w P{70FLP; ry+}3F; Sco/SM6; 3iso [a]	-	2Rc—2Rt
w^{1118}*; P{RS3}CB-0236-3, P{RS5}5-HA-1995* [c]	REC	2Rc—2Rt
w^{1118}*; P{RS3r}CB-0236-3, P{RS5r}5-HA-1995* [c]	SIM1, SIM2, SIM3	2Rc—2Rt
w^{1118}*; In(2R)51F11-56E2* [c]	INV1, INV2	2Rc—51F11|56E2-51F11|56E2—2Rt
w^{1118}*; P{RS3r}CB-0236-3, P{RS5r}5-HA-1995* [c]	REV1, REV2	2Rc—2Rt

[a] Department of Genetics, University of Cambridge [1]. [b] Szeged *Drosophila* Stock Center. [c] Present work.

Supporting References

1. Ryder E, Ashburner M, Bautista-Llacer R, Drummond J, Webster J, et al. (2007) The DrosDel deletion collection: a Drosophila genomewide chromosomal deficiency resource. Genetics 177: 615-629.
