# Supplementary material for: Evaluation of the Role of Functional Constraints on the Integrity of an Ultraconserved Region in the Genus Drosophila
Source: PLoS Genet. 2012 Feb 2;8(2):e1002475. doi: 10.1371/journal.pgen.1002475 (PMC3271063; doi:10.1371/journal.pgen.1002475)
Supplement: Table S5 — Primers used. (PDF) [file pgen.1002475.s024.pdf]

Table S5. Primers used

Primer	Sequence	Location
Plac1 [a]	CACCCAAGGCTCTGCTCCCACAAT	*P* element 5’ end, outwards orientation
Pry4 [a]	CAATCATATCGCTGTCTCACTCA	*P* element 3’ end, outwards orientation
w11678U [b]	TCATCGCAGATCAGAAGCGG	3’ exon of the reporter gene mini-*white*
CB-0236-3up	ACACGCCGCAAGATGAATAC	*2R*:11260327..11260346 (+)
CB-0236-3down	GATGACCAATTTCCGTTTGG	*2R*:11261115..11261096 (-)
5-HA-1995up	AAATTTTTCTGCCTCGCAAA	*2R*:15613845..15613864 (+)
5-HA-1995down	TCGAGTGGAATGAGTTGTGG	*2R*:15614470..15614451 (-)

[a] http://www.fruitfly.org/about/methods/inverse.pcr.html. [b] [1].

Supporting References

1. Golic KG, Golic MM (1996) Engineering the Drosophila genome: chromosome rearrangements by design. Genetics 144: 1693-1711.
