# Supplementary material for: Evaluation of the Role of Functional Constraints on the Integrity of an Ultraconserved Region in the Genus Drosophila
Source: PLoS Genet. 2012 Feb 2;8(2):e1002475. doi: 10.1371/journal.pgen.1002475 (PMC3271063; doi:10.1371/journal.pgen.1002475)
Supplement: Table S6 — Amplicons used to confirm the molecular organization of the genomic regions corresponding to the breakpoints of the inversion In(2R)51F11-56E2. (PDF) [file pgen.1002475.s025.pdf]

Table S6. Amplicons used to confirm the molecular organization of the genomic regions corresponding to the breakpoints of the inversion *In(2R)51F11-56E2*

Amplicon [a]	Primers	Size (nt)	Purpose
A [b]	CB-0236-3up / CB-0236-3down	~700	Verification of flanking sequence at the outer breakpoint in strains w^{1118} and 5-HA-1995
B [b]	5-HA-1995up / 5-HA-1995down	~600	Verification of flanking sequence at the inner breakpoint in strains w^{1118} and CB-0236-3
C [b]	CB-0236-3up / Plac1	~600	Verification of upstream flanking sequence to *P{RS3}CB-0236-3* and its derivatives
D [b]	Pry4 / CB-0236-3down	~500	Verification of downstream flanking sequence to *P{RS3}CB-0236-3* and its derivatives
E [b]	5-HA-1995up / Plac1	~600	Verification of upstream flanking sequence to *P{RS5}5-HA-1995* and its derivatives
F [b]	Pry4 / 5-HA-1995down	~300	Verification of downstream flanking sequence to *P{RS5}5-HA-1995* and its derivatives
G [c]	CB-0236-3up / w11678U	~3,400	Diagnostic presence of a reporter gene mini-*white* with two exons at the outer breakpoint in strains CB-0236-3 and REC
G´ [c]	CB-0236-3up / w11678U	~2,100	Diagnostic presence of a reporter gene mini-*white* with one exon in strains SIM and REV
H [c]	w11678U / 5-HA-1995down	~2,600	Diagnostic presence of a reporter gene mini-*white* with two exons at the inner breakpoint in strains 5-HA-1995, REC, and INV

[a] Figure S5 for physical location. [b] Amplified with Takara Taq. [c] Amplified with Takara Ex Taq.
