# Supplementary material for: Evaluation of the Role of Functional Constraints on the Integrity of an Ultraconserved Region in the Genus Drosophila
Source: PLoS Genet. 2012 Feb 2;8(2):e1002475. doi: 10.1371/journal.pgen.1002475 (PMC3271063; doi:10.1371/journal.pgen.1002475)
Supplement: Table S7 — Test for differences in progeny size among the strains generated in the course of the experiments using homozygous crosses. (PDF) [file pgen.1002475.s026.pdf]

Table S7. Test for differences in progeny size among the strains generated in the course of the experiments using homozygous crosses

		P [b]						
**Strain**	**Progeny Size** [a]	**INV2**	**REC**	**SIM1**	**SIM2**	**SIM3**	**REV1**	**REV2**
**INV1**	405.400, (345.635, 465.165)	0.9999	0.9156	0.9818	1.0000	0.9156	0.8276	0.9156
**INV2**	375.200, (281.541, 468.859)		0.7061	1.0000	0.9911	1.0000	1.0000	1.0000
**REC**	456.400, (346.495, 566.305)			0.9156	0.9911	0.7061	0.7061	0.8276
**SIM1**	379.400, (322.523, 436.277)				0.9156	0.9911	0.9999	0.9985
**SIM2**	412.200, (345.436, 478.964)					0.7061	0.9156	0.8276
**SIM3**	343.600, (280.041, 407.159)						1.0000	1.0000
**REV1**	363.600, (287.998, 439.202)							1.0000
**REV2**	359.600, (308.333, 410.867)							

[a] Mean, 95% CI (lower boundary, upper boundary). [b] According to Steel-Dwass test. $n = 5$ across strains.
