# Supplementary material for: Evaluation of the Role of Functional Constraints on the Integrity of an Ultraconserved Region in the Genus Drosophila
Source: PLoS Genet. 2012 Feb 2;8(2):e1002475. doi: 10.1371/journal.pgen.1002475 (PMC3271063; doi:10.1371/journal.pgen.1002475)
Supplement: Table S8 — Test for differences in sex ratio among the strains generated in the course of the experiments using homozygous crosses. (PDF) [file pgen.1002475.s027.pdf]

Table S8. Test for differences in sex ratio among the strains generated in the course of the experiments using homozygous crosses

		P [b]						
**Strain**	**Sex Ratio** [a]	**INV2**	**REC**	**SIM1**	**SIM2**	**SIM3**	**REV1**	**REV2**
**INV1**	-0.075, (-0.200, 0.050)	0.9999	0.9676	0.9999	1.0000	0.7061	1.0000	0.8276
**INV2**	-0.056, (-0.181, 0.069)		0.9985	1.0000	1.0000	1.0000	1.0000	0.7061
**REC**	0.029, (-0.096, 0.153)			0.9676	0.9911	1.0000	0.9676	0.1924
**SIM1**	-0.044, (-0.169, 0.081)				1.0000	0.9156	0.9999	0.5647
**SIM2**	-0.049, (-0.174, 0.076)					0.9911	1.0000	0.4220
**SIM3**	0.055, (-0.070, 0.180)						0.7061	0.1924
**REV1**	-0.062, (-0.187, 0.063)							0.4220
**REV2**	-0.280, (-0.405, -0.155)							

[a] Mean of log2 values, 95% CI (lower boundary, upper boundary). [b] According to Steel-Dwass test. $n = 5$ across strains.
