# Supplementary material for: Evaluation of the Role of Functional Constraints on the Integrity of an Ultraconserved Region in the Genus Drosophila
Source: PLoS Genet. 2012 Feb 2;8(2):e1002475. doi: 10.1371/journal.pgen.1002475 (PMC3271063; doi:10.1371/journal.pgen.1002475)
Supplement: Table S9 — Evaluation of the effect of the disrupted ultraconserved region CG15121–CG16894 in heterozygosis on a variety of traits. (PDF) [file pgen.1002475.s028.pdf]

Table S9. Evaluation of the effect of the disrupted ultraconserved region *CG15121-CG16894* in heterozygosis on a variety of traits

**Trait**	**Contrast**	***n***	**Statistical Test**	**D.F.**	***P***
Progeny size [a]					
Females	INV-1, INV-2, REC	10-15	ANOVA	2, 32	0.1053
Males	INV-1, INV-2, REC	10-15	ANOVA	2, 32	0.3556
Total	INV-1, INV-2, REC	10-15	ANOVA [c]	2, 19.52	0.1301
Female to male ratio [a, b]	INV-1, INV-2, REC	10-15	ANOVA	2, 32	0.1111
Mendelian ratios (1:2:1)					
Females	REC/SIM1	5	*G*-test	2	0.1691
	INV1/SIM1	5	*G*-test	2	0.8908
	INV2/SIM1	5	*G*-test	2	0.1026
	REC/REV1	5	*G*-test	2	0.5663
	INV1/REV1	5	*G*-test	2	0.3147
	REC/REV2	5	*G*-test	2	0.5233
	INV2/REV2	5	*G*-test	2	0.8822
Males	REC/SIM1	5	*G*-test	2	0.8969
	INV1/SIM1	5	*G*-test	2	0.8635
	INV2/SIM1	5	*G*-test	2	0.2068
	REC/REV1	5	*G*-test	2	0.6153
	INV1/REV1	5	*G*-test	2	0.6434
	REC/REV2	5	*G*-test	2	0.2708
	INV2/REV2	5	*G*-test	2	0.1055
Total	REC/SIM1	5	*G*-test	2	0.2719
	INV1/SIM1	5	*G*-test	2	0.8321
	INV2/SIM1	5	*G*-test	2	0.0908
	REC/REV1	5	*G*-test	2	0.7035
	INV1/REV1	5	*G*-test	2	0.6829
	REC/REV2	5	*G*-test	2	0.1723
	INV2/REV2	5	*G*-test	2	0.2331

[a] Upon pooling the data from the various genetic backgrounds in which the chromosomes tested (REC, INV1, INV2) were in heterozygosis (SIM1, REV1, and REV2). [b] Upon log transformation. [c] Welch's ANOVA.
