# Supplementary material for: Evaluation of the Role of Functional Constraints on the Integrity of an Ultraconserved Region in the Genus Drosophila
Source: PLoS Genet. 2012 Feb 2;8(2):e1002475. doi: 10.1371/journal.pgen.1002475 (PMC3271063; doi:10.1371/journal.pgen.1002475)
Supplement: Table S10 — Evaluation of the effect of the disrupted ultraconserved region CG15121–CG16894 on relative viability in competition experiments prior to imago emergence. (PDF) [file pgen.1002475.s029.pdf]

Table S10. Evaluation of the effect of the disrupted ultraconserved region *CG15121-CG16894* on relative viability in competition experiments prior to imago emergence

**Starting Proportion** [a]	**Experiment**	**Tested Chromosome**	**Condition** [b]	**Relative Viability** [c]	**Contrast Chromosomes**	**Contrast Statistical Test** [d]	**Contrast *P***
1:3	SIM1/SIM1 vs REC/SIM1	REC	Het	0.578, (0.141, 0.843)	REC, INV1	Tukey-Kramer HSD	<0.0001
	SIM1/SIM1 vs INV1/SIM1	INV1	Het	1.445, (0.227, 0.929)	REC, INV2	Tukey-Kramer HSD	0.9329
	SIM1/SIM1 vs INV2/SIM1	INV2	Het	-0.600, (-1.818, -1.116)	INV1, INV2	Tukey-Kramer HSD	<0.0001
	SIM1/SIM1 vs REC/REC	REC	Hom	0.041, (-0.230, 0.312)	REC, INV1	Tukey-Kramer HSD	1
	SIM1/SIM1 vs INV1/INV1	INV1	Hom	0.040, (-0.231, 0.311)	REC, INV2	Tukey-Kramer HSD	0.0051
	SIM1/SIM1 vs INV2/INV2	INV2	Hom	0,685, (0.414, 0.956)	INV1, INV2	Tukey-Kramer HSD	0.0051
	REV1/REV1 vs REC/REV1	REC	Het	-0.512, (-0.892, -0.132)	REC, INV1	*t*-test	0.2341
	REV1/REV1 vs INV1/REV1	INV1	Het	-0.827, (-1.207, -0.447)			
	REV1/REV1 vs REC/REC	REC	Hom	0.892, (0.696, 1.088)	REC, INV1	*t*-test	0.0002
	REV1/REV1 vs INV1/INV1	INV1	Hom	0.288, (0.092, 0.484)			
	REV2/REV2 vs REC/REV2	REC	Het	0.583, (0.267, 0.899)	REC, INV2	*t*-test	0.1003
	REV2/REV2 vs INV2/REV2	INV2	Het	0.952, (0.636, 1.268)			
	REV2/REV2 vs REC/REC	REC	Hom	0.257, (0.074, 0.441)	REC, INV2	*t*-test	0.3068
	REV2/REV2 vs INV2/INV2	INV2	Hom	0.387, (0.203, 0.571)			

Table S10. Evaluation of the effect of the disrupted ultraconserved region *CG15121-CG16894* on relative viability in competition experiments prior to imago emergence

Starting Proportion [a]	Experiment	Tested Chromosome	Condition [b]	Relative Viability [c]	Contrast: Chromosomes	Contrast: Statistical Test [d]	Contrast: *P*
1:1	SIM1/SIM1 vs REC/SIM1	REC	Het	0.484, (0.145, 0.823)	REC, INV1	Tukey-Kramer HSD	0.0014
	SIM1/SIM1 vs INV1/SIM1	INV1	Het	-0.439, (-0.778, -0.100)	REC, INV2	Tukey-Kramer HSD	0.2483
	SIM1/SIM1 vs INV2/SIM1	INV2	Het	0.102, (-0.237, 0.441)	INV1, INV2	Tukey-Kramer HSD	0.0705
	SIM1/SIM1 vs REC/REC	REC	Hom	0.233, (-0.017, 0.483)	REC, INV1	Tukey-Kramer HSD	0.0035
	SIM1/SIM1 vs INV1/INV1	INV1	Hom	-0.213, (-0.463, 0.037)	REC, INV2	Tukey-Kramer HSD	0.0035
	SIM1/SIM1 vs INV2/INV2	INV2	Hom	0.853, (0.603, 1.103)	INV1, INV2	Tukey-Kramer HSD	<0.0001
	REV1/REV1 vs REC/REV1	REC	Het	-0.412, (-0.642, -0.182)	REC, INV1	*t*-test	0.0208
	REV1/REV1 vs INV1/REV1	INV1	Het	0.805, (-1.035, -0.575)			
	REV1/REV1 vs REC/REC	REC	Hom	0.715, (0.336, 1.094)	REC, INV1	*t*-test	0.1027
	REV1/REV1 vs INV1/INV1	INV1	Hom	0.276, (-0.103, 0.655)			
	REV2/REV2 vs REC/REV2	REC	Het	0.547, (0.305, 0.789)	REC, INV2	*t*-test	0.0747
	REV2/REV2 vs INV2/REV2	INV2	Het	0.855, (0.613, 1.097)			
	REV2/REV2 vs REC/REC	REC	Hom	0.337, (0.084, 0.590)	REC, INV2	*t*-test	0.1436
	REV2/REV2 vs INV2/INV2	INV2	Hom	0.597, (0.344, 0850)			

Table S10. Evaluation of the effect of the disrupted ultraconserved region *CG15121-CG16894* on relative viability in competition experiments prior to imago emergence

Starting Proportion [a]	Experiment	Tested Chromosome	Condition [b]	Relative Viability [c]	Contrast Chromosomes	Contrast Statistical Test [d]	Contrast *P*
3:1	SIM1/SIM1 vs REC/SIM1	REC	Het	0.527, (0.267, 0787)	REC, INV1	Tukey-Kramer HSD	0.0010
	SIM1/SIM1 vs INV1/SIM1	INV1	Het	-0.204, (-0.464, 0.056)	REC, INV2	Tukey-Kramer HSD	0.1185
	SIM1/SIM1 vs INV2/SIM1	INV2	Het	0.159, (-0.101, 0.419)	INV1, INV2	Tukey-Kramer HSD	0.1249
	SIM1/SIM1 vs REC/REC	REC	Hom	0.310, (0.072, 0.549)	REC, INV1	Tukey-Kramer HSD	0.8891
	SIM1/SIM1 vs INV1/INV1	INV1	Hom	0.234, (-0.004, 0.472)	REC, INV2	Tukey-Kramer HSD	0.0006
	SIM1/SIM1 vs INV2/INV2	INV2	Hom	1.016, (0.778, 1.254)	INV1, INV2	Tukey-Kramer HSD	0.0002
	REV1/REV1 vs REC/REV1	REC	Het	-0.610, (-1.067, -0.645)	REC, INV1	*t*-test	0.0983
	REV1/REV1 vs INV1/REV1	INV1	Het	-0.857, (-0.820, -0.400)			
	REV1/REV1 vs REC/REC	REC	Hom	0.715, (0.452, 0.978)	REC, INV1	*t*-test	0.0026
	REV1/REV1 vs INV1/INV1	INV1	Hom	0.097, (-0.166, 0.360)			
	REV2/REV2 vs REC/REV2	REC	Het	-0.255, (0.740, 1.312)	REC, INV2	*t*-test	<0.0001
	REV2/REV2 vs INV2/REV2	INV2	Het	1.026, (-0.541, 0.031)			
	REV2/REV2 vs REC/REC	REC	Hom	0.051, (-0.142, 0.244)	REC, INV2	*t*-test	0.0022
	REV2/REV2 vs INV2/INV2	INV2	Hom	0.514, (0.321, 0707)			

[a] Tester embryo : embryo carrying the tested chromosome. [b] Het, heterozygosis; Hom, homozygosis. [c] Mean, 95% CI (lower boundary, upper boundary). [d] In the case of *t*-tests, d.f. = 18. Relative viabilities were log2-transformed. *n* = 10 for all experiments.
