# Supplementary material for: Evaluation of the Role of Functional Constraints on the Integrity of an Ultraconserved Region in the Genus Drosophila
Source: PLoS Genet. 2012 Feb 2;8(2):e1002475. doi: 10.1371/journal.pgen.1002475 (PMC3271063; doi:10.1371/journal.pgen.1002475)
Supplement: Table S11 — Fertility of males carrying the ultraconserved region CG15121–CG1689 in its disrupted or intact form. (PDF) [file pgen.1002475.s030.pdf]

Table S11. Fertility of males carrying the ultraconserved region *CG15121-CG1689* in its disrupted or intact form

		Contrast [b]	
**Tested Chromosome**	**Progeny Size [a]**	**Chromosomes**	***P***
REC	115.680, (98.84, 132.52)	REC, INV1	0.4200
INV1	102.813, (81.77, 123.86)	REC, INV2	0.2616
INV2	133.333, (113.49, 153.18)	INV1, INV2	0.0215

[a] Mean, 95% CI (lower boundary, upper boundary). [b] According to the Steel-Dwass test. n = 10 for each tested chromosome.
