# Supplementary material for: Evaluation of the Role of Functional Constraints on the Integrity of an Ultraconserved Region in the Genus Drosophila
Source: PLoS Genet. 2012 Feb 2;8(2):e1002475. doi: 10.1371/journal.pgen.1002475 (PMC3271063; doi:10.1371/journal.pgen.1002475)
Supplement: Table S12 — Sperm competence of males carrying the ultraconserved region CG15121–CG1689 in its disrupted or intact form in double mating experiments. (PDF) [file pgen.1002475.s031.pdf]

Table S12. Sperm competence of males carrying the ultraconserved region *CG15121-CG1689* in its disrupted or intact form in double mating experiments

**Mating Experiment**	**Tested Chromosome**	***n***	**Fraction of w^+ offspring** [a]
Direct	REC	22	0.410, (0.3032, 0.5168)
	INV1	10	0.422, (0.2636, 0.5804)
	INV2	14	0.451, (0.3168, 0.5846)
Reciprocal	REC	25	0.614, (0.5196, 0.7084)
	INV1	16	0.573, (0.4552, 0.692)
	INV2	14	0.607, (0.4810, 0.7332)

[a] Mean, 95% CI (lower boundary, upper boundary).
