# Supplementary material for: Evaluation of the Role of Functional Constraints on the Integrity of an Ultraconserved Region in the Genus Drosophila
Source: PLoS Genet. 2012 Feb 2;8(2):e1002475. doi: 10.1371/journal.pgen.1002475 (PMC3271063; doi:10.1371/journal.pgen.1002475)
Supplement: Table S13 — Mating ability of flies carrying the ultraconserved region CG15121–CG1689 in its disrupted or intact form in three different timeframes. (PDF) [file pgen.1002475.s032.pdf]

Table S13. Mating ability of flies carrying the ultraconserved region *CG15121-CG1689* in its disrupted or intact form in three different timeframes

Timeframe	Tested Chromosome	Females Fertilized [a]
1 h	REC	1.2, (0.6745, 1.7253)
	INV1	1.5, (0.9745, 2.0253)
	INV2	0.8, (0.2746, 1.3253)
3 h	REC	1.9, (1.3717, 2.4283)
	INV1	2.3, (1.7717, 2.8283)
	INV2	2.1, (1.5717, 2.6283)
6 h	REC	3.5, (2.539, 4.4361)
	INV1	3.2, (2.2639, 4.1361)
	INV2	3.3, (2.3639, 4.2361)

[a] Mean, 95% CI (lower boundary, upper boundary). *n* = 10 for each tested chromosome.
