# Supplementary material for: Evaluation of the Role of Functional Constraints on the Integrity of an Ultraconserved Region in the Genus Drosophila
Source: PLoS Genet. 2012 Feb 2;8(2):e1002475. doi: 10.1371/journal.pgen.1002475 (PMC3271063; doi:10.1371/journal.pgen.1002475)
Supplement: Table S14 — Response of flies carrying the ultraconserved region CG15121–CG1689 in its disrupted or intact form to a variety of volatile compounds. (PDF) [file pgen.1002475.s033.pdf]

Table S14. Response of flies carrying the ultraconserved region *CG15121-CG1689* in its disrupted or intact form to a variety of volatile compounds

**Volatile Compound**	**Tested Chromosome**	**Avoidance Score** [a]	**Contrast** [b]	
			**Chromosomes**	***P***
Water				
Females	REC	1.75, (1.3637, 2.1363)	REC, INV1	0.0241
	INV1	2.40, (2.1454, 2.6546)	REC, INV2	0.0360
	INV2	2.39, (2.1130, 2.6670)	INV1, INV2	1
Males	REC	2.32, (1.9987, 2.6413)	REC, INV1	0.9718
	INV1	2.49, (1.9173, 3.0627)	REC, INV2	0.8163
	INV2	2.22, (1.8882, 2.5518)	INV1, INV2	0.7942
Ethanol 10^{-3}				
Females	REC	1.21, (0.8033, 1.6167)	REC, INV1	0.0191
	INV1	2.04, (1.6421, 2.4379)	REC, INV2	0.0155
	INV2	1.98, (1.6587, 2.3013)	INV1, INV2	1
Males	REC	1.83, (1.4197, 2.2403)	REC, INV1	0.0593
	INV1	1.13, (0.6196, 1.6404)	REC, INV2	0.9874
	INV2	1.84, (1.3924, 2.2876)	INV1, INV2	0.0331
Ethanol $10^{-0.5}$				
Females	REC	1.68, (1.2929, 2.0671)	REC, INV1	0.1083
	INV1	2.14, (1.7295, 2.5505)	REC, INV2	0.0267
	INV2	2.25, (1.9971, 2.5029)	INV1, INV2	0.9616
Males	REC	2.61, (2.2384, 2.9816)	REC, INV1	0.3779
	INV1	2.22, (1.8100, 2.6300)	REC, INV2	0.5819
	INV2	2.20, (1.5349, 2.8651)	INV1, INV2	0.9874
Acetone $10^{-4.5}$				
Females	REC	1.89, (1.6535, 2.1365)	REC, INV1	0.0007
	INV1	2.85, (2.5971, 3.1029)	REC, INV2	0.1854
	INV2	2.22, (1.9658, 2.4742)	INV1, INV2	0.0086

Table S14. Response of flies carrying the ultraconserved region *CG15121-CG1689* in its disrupted or intact form to a variety of volatile compounds

**Volatile Compound**	**Tested Chromosome**	**Avoidance Score** [a]	**Contrast** [b] **Chromosomes**	***P***
Males	REC	2.35, (2.0555, 2.6445)	REC, INV1	0.0268
	INV1	2.88, (2.6646, 3.0954)	REC, INV2	0.0714
	INV2	2.88, (2.4827, 3.2773)	INV1, INV2	1
Acetone $10^{-1.5}$				
Females	REC	2.54, (2.1436, 2.9364)	REC, INV1	0.4231
	INV1	2.91, (2.4483, 3.3717)	REC, INV2	0.9616
	INV2	2.64, (2.1689, 3.1111)	INV1, INV2	0.3389
Males	REC	3.13, (2.6627, 3.5973)	REC, INV1	0.9968
	INV1	2.97, (2.4942, 3.4458)	REC, INV2	0.5144
	INV2	2.80, (2.5099, 3.0901)	INV1, INV2	0.4908
Benzaldehyde $10^{-3.5}$				
Females	REC	2.33, (1.8185, 2.8415)	REC, INV1	0.1704
	INV1	2.66, (2.4384, 2.8816)	REC, INV2	0.7495
	INV2	2.43, (2.2867, 2.5733)	INV1, INV2	0.2432
Males	REC	2.80, (2.3603, 3.2397)	REC, INV1	0.9620
	INV1	2.80, (2.1375, 3.4625)	REC, INV2	0.1889
	INV2	2.33, (1.9440, 2.7160)	INV1, INV2	0.3807
Benzaldehyde $10^{-0.5}$				
Females	REC	4.64, (4.1328, 5.1472)	REC, INV1	0.5390
	INV1	4.85, (4.7657, 4.9343)	REC, INV2	0.4020
	INV2	4.85, (4.7805, 4.9195)	INV1, INV2	0.9991
Males	REC	4.85, (4.7059, 4.9941)	REC, INV1	1
	INV1	4.78, (4.4923, 5.0677)	REC, INV2	0.4251
	INV2	4.72, (4.5298, 4.9102)	INV1, INV2	0.5217

[a] Mean, 95% CI (lower boundary, upper boundary). [b] According to the Steel-Dwass test. n = 10 for each volatile compound by sex combination assayed.
