# Supplementary material for: Evaluation of the Role of Functional Constraints on the Integrity of an Ultraconserved Region in the Genus Drosophila
Source: PLoS Genet. 2012 Feb 2;8(2):e1002475. doi: 10.1371/journal.pgen.1002475 (PMC3271063; doi:10.1371/journal.pgen.1002475)
Supplement: Table S15 — Performance of strains carrying the ultraconserved region CG15121–CG1689 in its disrupted or intact form based on four proxies of global homeostasis. (PDF) [file pgen.1002475.s034.pdf]

Table S15. Performance of strains carrying the ultraconserved region *CG15121-CG1689* in its disrupted or intact form based on four proxies of global homeostasis

	Strains [a]		
**Proxy (Parameter Measured)**	**REC**	**INV1**	**INV2**
Negative gravitaxis (height reached)			
Females	0.812, (0.677, 0.946)	0.844, (0.768, 0.920)	0.834, (0.753, 0.915)
Males	0.772, (0.674, 0.870)	0.764, (0.672, 0856)	0.716, (0.610, 0.821)
Heat-shock resistance (fraction surviving flies)			
Females	0.880, (0.776, 0.984)	0.860, (0.667, 1.053)	0.700, (0.437, 096)
Males	0.610, (0.499, 0.721)	0.700, (0.584, 0.816)	0.690, (0.518, 0.86)
Desiccation resistance (time to death)			
Females	17.15, (16.073, 18.227)	16.95, (15.731, 18.169)	17.10, (15.905, 18.295)
Males	12.75, (11.948, 13.552)	13.05, (12.453, 13.647)	13.05, (12.493, 13.607)
Starvation resistance (time to death)			
Females	2.10, (1.905, 2.295)	2.175, (1.932, 2.418)	2.625, (2.496, 2.754)
Males	2.17, (2.001, 2.349)	2.125, (2.021, 2.229)	2.175, (2.001, 2.349)

[a] Mean, 95% CI (lower boundary, upper boundary). $n = 20$ for all proxies except for heat-shock ($n = 5$).
