# Supplementary material for: Evaluation of the Role of Functional Constraints on the Integrity of an Ultraconserved Region in the Genus Drosophila
Source: PLoS Genet. 2012 Feb 2;8(2):e1002475. doi: 10.1371/journal.pgen.1002475 (PMC3271063; doi:10.1371/journal.pgen.1002475)
Supplement: Table S16 — Expression differences among strains using a one-way ANOVA at FDR 0.01. (PDF) [file pgen.1002475.s035.pdf]

Table S16. Expression differences among strains using a one-way ANOVA at FDR 0.01

	REC	INV1	INV2	SIM1	REV1	REV2
REC	-	0 / 2	0 / 9	2 / 5	2 / 14	2 / 44
INV1		-	0 / 3	2 / 7	6 / 11	2 / 11
INV2			-	2 /8	3 / 5	2 / 5
SIM1				-	2 /3	0 /2
REV1					-	0 / 0
REV2						-

Males / females.
