# Supplementary material for: Evaluation of the Role of Functional Constraints on the Integrity of an Ultraconserved Region in the Genus Drosophila
Source: PLoS Genet. 2012 Feb 2;8(2):e1002475. doi: 10.1371/journal.pgen.1002475 (PMC3271063; doi:10.1371/journal.pgen.1002475)
Supplement: Table S17 — Expression differences detected in six planned contrasts using one-way ANOVA at FDR 0.01. (PDF) [file pgen.1002475.s036.pdf]

Table S17. Expression differences detected in six planned contrasts using one-way ANOVA at FDR 0.01

Planned Contrast	Males	Females
INV1 vs INV2 [a]	0	3
REV1 vs REV2 [a]	0	0
SIM vs (REV1, REV2) [b]	0	1
REC vs (SIM1, REV1, REV2) [b]	3	13
REC vs (INV1, INV2) [b]	0	1
(INV1, INV2) vs (SIM1, REV1, REV2) [b]	5	114

[a] Dataset S1. [b] Dataset S2.
