# Supplementary material for: Evaluation of the Role of Functional Constraints on the Integrity of an Ultraconserved Region in the Genus Drosophila
Source: PLoS Genet. 2012 Feb 2;8(2):e1002475. doi: 10.1371/journal.pgen.1002475 (PMC3271063; doi:10.1371/journal.pgen.1002475)
Supplement: Table S18 — Statistically significant enrichment for biological coherent patterns (GO term ontology, KEGG pathway, and Interpro domains) among genes differentially expressed in females in at least one of the six planned contrasts. (PDF) [file pgen.1002475.s037.pdf]

Table S18. Statistically significant enrichment for biological coherent patterns (GO term ontology, KEGG pathway, and Interpro domains) among genes differentially expressed in females in at least one of the six planned contrasts

**Annotation Cluster**	**Term** [a]	**Contributing Genes** [b]	**Percent**	***P* value** [c]
1	GO:0007601~visual perception (*bp*)	9	8.490566	2.08E-05
	GO:0050953~sensory perception of light stimulus (*bp*)	9	8.490566	1.55E-05
	GO:0007600~sensory perception (*bp*)	11	10.377358	0.0018081
2	GO:0001505~regulation of neurotransmitter levels (*bp*)	9	8.490566	2.34E-04
	GO:0007268~synaptic transmission (*bp*)	10	9.4339623	9.42E-04
	GO:0019226~transmission of nerve impulse (*bp*)	10	9.4339623	9.89E-04
	GO:0007267~cell-cell signalling (*bp*)	10	9.4339623	0.0013774
3	GO:0007603~phototransduction, visible light (*bp*)	6	5.6603774	4.44E-05
	GO:0009584~detection of visible light (*bp*)	6	5.6603774	1.56E-04
	GO:0022400~regulation of rhodopsin mediated signalling pathway (*bp*)	5	4.7169811	2.97E-04
	GO:0016059~deactivation of rhodopsin mediated signalling (*bp*)	5	4.7169811	2.97E-04
	GO:0009586~rhodopsin mediated phototransduction (*bp*)	5	4.7169811	4.66E-04

Table S18. Statistically significant enrichment for biological coherent patterns (GO term ontology, KEGG pathway, and Interpro domains) among genes differentially expressed in females in at least one of the six planned contrasts

Annotation Cluster	Term [a]	Contributing Genes [b]	Percent	*P* value [c]
	GO:0016056~rhodopsin mediated signalling pathway (*bp*)	5	4.7169811	5.38E-04
	GO:0050908~detection of light stimulus involved in visual reception (*bp*)	5	4.7169811	9.15E-04
	GO:0008277~regulation of G-protein coupled receptor protein signalling pathway (*bp*)	5	4.7169811	9.63E-04
	GO:0050962~detection of light stimulus involved in sensory perception (*bp*)	5	4.7169811	9.63E-04
	GO:0007602~phototransduction (*bp*)	6	5.6603774	9.92E-04
	GO:0009583~detection of light stimulus (*bp*)	6	5.6603774	0.0015475
	GO:0009582~detection of abiotic stimulus (*bp*)	6	5.6603774	0.0022066
	GO:0009581~detection of external stimulus (*bp*)	6	5.6603774	0.0031789
	GO:0009416~response to light stimulus (*bp*)	7	6.6037736	0.0035542
	GO:0009314~response to radiation (*bp*)	7	6.6037736	0.0061349
	GO:0050906~detection of stimulus involved in sensory perception (*bp*)	5	4.7169811	0.0123216
	GO:0051606~detection of stimulus (*bp*)	6	5.6603774	0.0215457
	GO:0009628~response to abiotic stimulus (*bp*)	8	7.5471698	0.0263414

Table S18. Statistically significant enrichment for biological coherent patterns (GO term ontology, KEGG pathway, and Interpro domains) among genes differentially expressed in females in at least one of the six planned contrasts

Annotation Cluster	Term [a]	Contributing Genes [b]	Percent	*P* value [c]
4	GO:0042133~neurotransmitter metabolic process (*bp*)	4	3.7735849	9.80E-04
	GO:0042136~neurotransmitter biosynthetic process (*bp*)	3	2.8301887	0.011968
5	GO:0042133~neurotransmitter metabolic process (*bp*)	4	3.7735849	9.80E-04
	GO:0008291~acetylcholine metabolic process (*bp*)	3	2.8301887	0.0043694
	GO:0042439~ethanolamine and derivative metabolic process (*bp*)	3	2.8301887	0.0329842
6	GO:0042398~cellular amino acid derivative biosynthetic process (*bp*)	5	4.7169811	0.0014427
	GO:0006576~biogenic amine metabolic process (*bp*)	5	4.7169811	0.0039768
7	GO:0045202~synapse (*cc*)	7	6.6037736	0.0482615
	GO:0030054~cell junction (*cc*)	7	6.6037736	0.0416167

Table S18. Statistically significant enrichment for biological coherent patterns (GO term ontology, KEGG pathway, and Interpro domains) among genes differentially expressed in females in at least one of the six planned contrasts

**Annotation Cluster**	**Term** [a]	**Contributing Genes** [b]	**Percent**	***P* value** [c]
8	GO:0048066~pigmentation during development (*bp*)	5	4.7169811	0.024407
	GO:0043473~pigmentation (*bp*)	5	4.7169811	0.0274781
	GO:0006726~eye pigment biosynthetic process (*bp*)	4	3.7735849	0.0392668
	GO:0042441~eye pigment metabolic process (*bp*)	4	3.7735849	0.0414843
9	GO:0006887~exocytosis (*bp*)	5	4.716981	0.0315

Results according to DAVID [1]. [a] *bp* (biological process) and *cc* (cellular component) refer to different GO term categories. [b] Number of genes contributing to a particular functional class. [c] After Benjamini-Hochberg correction.

Supporting References

1. Huang da W, Sherman BT, Lempicki RA (2009) Systematic and integrative analysis of large gene lists using DAVID bioinformatics resources. Nat Protoc 4: 44-57.
