# Supplementary material for: Evaluation of the Role of Functional Constraints on the Integrity of an Ultraconserved Region in the Genus Drosophila
Source: PLoS Genet. 2012 Feb 2;8(2):e1002475. doi: 10.1371/journal.pgen.1002475 (PMC3271063; doi:10.1371/journal.pgen.1002475)
Supplement: Table S19 — Ability to detect significant differences between flies carrying the ultraconserved region CG15121–CG1689 in its disrupted or intact form across some of the experiments performed. (PDF) [file pgen.1002475.s038.pdf]

Table S19. Ability to detect significant differences between flies carrying the ultraconserved region *CG15121-CG1689* in its disrupted or intact form across some of the experiments performed

	Sample Size		
**Experiment** [a]	**Actual**	**Power = 0.5** [b]	**Power = 0.8** [b]
Heterozygous crosses (male progeny)	35	42	78
Heterozygous crosses (sex ratio)	35	42	75
Heterozygous crosses (Mendelian proportions) [c]	712	794	1,543
Mating ability (1 h)	30	45	81
Mating ability (6 h)	30	666	1,293
Sperm competition (reciprocal crosses)	55	996	1,932
Negative gravitaxis (males)	60	330	636
Heat-shock resistance (females)	15	21	39
Desiccation resistance (females)	60	223,374	434,181

[a] Carriers of chromosomes with the ultraconserved region in its disrupted form (INV1, INV2) showed lower values than the carriers of the ultraconserved region in its intact form (REC) but the difference was not statistically significant at $\alpha = 0.05$, samples size used, variances associated with the traits analyzed, and power achieved. [b] As estimated with GPower 3.1.3 [1]. [c] For the male progeny of heterozygous crosses involving INV2/REV2, the closest case to show statistically significant differences from the expected Mendelian ratios ($P = 0.1055$).

Supporting References

1. Faul F, Erdfelder E, Lang AG, Buchner A (2007) G*Power 3: a flexible statistical power analysis program for the social, behavioral, and biomedical sciences. Behav Res Methods 39: 175-191.
