# Supplementary material for: Evaluation of the Role of Functional Constraints on the Integrity of an Ultraconserved Region in the Genus Drosophila
Source: PLoS Genet. 2012 Feb 2;8(2):e1002475. doi: 10.1371/journal.pgen.1002475 (PMC3271063; doi:10.1371/journal.pgen.1002475)
Supplement: Table S20 — Number of tRNA, rRNA, and snoRNA genes in the ultraconserved region CG15121–CG16894 with potential to mediate NAHR events. (PDF) [file pgen.1002475.s039.pdf]

Table S20. Number of tRNA, rRNA, and snoRNA genes in the ultraconserved region *CG15121-CG16894* with potential to mediate NAHR events

**Species (release)** [a]	**tRNA genes**	**rRNA genes**	**snoRNA**
D. melanogaster (5.31)	9	104	1
D. yakuba (1.3)	10	15	1
D. erecta (1.3)	8	17	1
D. ananassae (1.3)	9	0	0
D. pseudoobscura (2.21)	6	0	0
D. willistoni (1.3)	17	0	0
D. virilis (1.2)	4	3	0
D. mojavensis (1.3)	7	0	0
D. grimshawi (1.3)	5	0	0

Between *CG8517* and *CG12501* [1]; pseudogenes are also included.

[a] Those previously examined [2] and sorted by their phylogenetic distance to *D. melanogaster*.

Supporting References

1. Tweedie S, Ashburner M, Falls K, Leyland P, McQuilton P, et al. (2009) FlyBase: enhancing Drosophila Gene Ontology annotations. Nucleic Acids Res 37: D555-559.

2. von Grotthuss M, Ashburner M, Ranz JM (2010) Fragile regions and not functional constraints predominate in shaping gene organization in the genus Drosophila. Genome Res 20: 1084-1096.
